# Electrically Conductive Materials: Opportunities and Challenges in Tissue Engineering

**DOI:** 10.3390/biom9090448

**Published:** 2019-09-04

**Authors:** Azadeh Saberi, Farzaneh Jabbari, Payam Zarrintaj, Mohammad Reza Saeb, Masoud Mozafari

**Affiliations:** 1 Nanotechnology and Advanced Materials Department, Materials and Energy Research Center (MERC), P.O. Box: 31787-316 Tehran, Iran (A.S.) (F.J.); 2 Polymer Engineering Department, Faculty of Engineering, Urmia University, P.O. Box: 5756151818-165 Urmia, Iran; 3 Department of Resin and Additives, Institute for Color Science and Technology, P.O. Box: 16765-654 Tehran, Iran; 4 Department of Tissue Engineering & Regenerative Medicine, Faculty of Advanced Technologies in Medicine, Iran University of Medical Sciences (IUMS), P.O Box: 14665-354 Tehran, Iran

**Keywords:** electrically conductive materials, cell response, biomaterials, nanomaterials, interface, tissue engineering, regenerative medicine

## Abstract

Tissue engineering endeavors to regenerate tissues and organs through appropriate cellular and molecular interactions at biological interfaces. To this aim, bio-mimicking scaffolds have been designed and practiced to regenerate and repair dysfunctional tissues by modifying cellular activity. Cellular activity and intracellular signaling are performances given to a tissue as a result of the function of elaborated electrically conductive materials. In some cases, conductive materials have exhibited antibacterial properties; moreover, such materials can be utilized for on-demand drug release. Various types of materials ranging from polymers to ceramics and metals have been utilized as parts of conductive tissue engineering scaffolds, having conductivity assortments from a range of semi-conductive to conductive. The cellular and molecular activity can also be affected by the microstructure; therefore, the fabrication methods should be evaluated along with an appropriate selection of conductive materials. This review aims to address the research progress toward the use of electrically conductive materials for the modulation of cellular response at the material-tissue interface for tissue engineering applications.

## 1. Introduction

According to statistics, only in the US, one person is listed as waiting for an organ transplant every fifteen minutes [1,2,3]. Unfortunately, less than half of the waiting patients are lucky enough to receive an appropriate organ from a pardoner due to exponential growth in the list of expectants. This raising dearth, however, is unable to be met by an accumulation of transplantable organs that has stagnated over the previous decade. One of the most undertaken strategies is tissue engineering, which reduces the organ shortage catastrophe thanks to artificial tissue design by the use of a combination of cells, engineering principles, and materials [4,5,6]. Tissue engineering techniques have been frequently applied to many types of tissues and organs such as skin, heart, muscle, nerve, bone, cartilage, and cornea [7,8,9,10]. In the body, tissue cells are besieged by a sophisticated mechanical, chemical, and electrical milieu. Commonly-used in vitro culture techniques have limited choices for mimicking all micro-environmental factors to direct stem cell differentiation in a developing organ [11,12,13]. Tissue properties such as stiffness and biosignals determine the cellular activity, including adhesion, proliferation, differentiation, and growth, that the architected scaffold should display to mimic the native tissue properties for damaged tissue to guarantee required regeneration. For instance, the stiffness of a scaffold is responsible for the formation of a brindled structure for skeletal myoblasts, stimulation of capillary tubes for endothelial cells, and neurite outgrowth for neuron cells [14]. Since cellular fate is modulated by cell-scaffold interactions, efforts have been done to regulate cellular responses by controlling the topography, 3D geometry, or chemical composition of cell substrates [15,16]. Additionally, some external factors can potentially affect cell–material interactions and biocompatibility including: Physical stimulation using surface topology; biochemical stimulations using release of growth factors; and mechanical and electrical stimulation (ES) [17,18,19]. The impact of electrical inducement on tissues has been defined since the 1960s when Bassett et al. proved that the electrical stimulation affects the bone formation [20]. It has been proved and explained how tissue microenvironment experiences a field of 1 V/cm during wound healing [21]. It has also been demonstrated that in vitro application of electrophysiologically DC fields (1–10 V/cm) and AC currents (10 to 100 mA) governs cellular behavior via interference in migration, cytoskeleton organization, alignment of neural cells, vascular endothelial, cardiofibroblasts, and myoblast cells, and enhances neurite outgrowth in nerve cells, differentiation, collagen production, and enhances calcification of osteoblasts [22,23]. Altogether, these preliminary studies have confirmed that the electrical and mechanical properties of scaffolds should be properly controlled for the development of physiologically healthy artificial tissues [24,25]. While there are several studies on conducting polymers for microelectronic and optoelectronic applications, researchers are exerting their focus toward biomedical applications; especially, biosensing, drug delivery, bioactuators, bioimaging and tissue engineering that benefit from developments in electroactive biomaterials [26,27,28]. The common attributes of conducting polymers (CPs) such as polyaniline (PANi), poly(3, 4-ethylenedioxythiophene) (PEDOT), and polypyrrole (PPy) are demanded for tissue engineering and regenerative medicine applications such as electroactivity, reversible oxidation, hydrophobicity, biocompatibility and surface topography. Nevertheless, elongated in vivo degradation time of conducting polymers may result in inflammation and requirement of surgical removal. To overcome such problems, researchers are now working on the development of biodegradable CPs [29,30].

## 2. Conductive Materials in Tissue Engineering

It is known that biomaterials surface properties have an important impact on cellular activities and cell–substrate interactions. The ability to keeping cells on the surface rather than within the hydrophobic scaffolds is one of the challenging issues in scaffold design. In this sense, surface treatment strategies have been developed to prepare substrates with high cell attachment potential. Along with the surface treatment, tissue engineering strategies can lead cells fate into particular, favorable lineages. Although electrical stimulation currents can be propagated via ionically conductive culture media, a more intended successful delivery requires electrical conductivity within three dimensional scaffolds for better tissue repair [31]. Electrical stimulation was revealed to have affirmative influences on the function and behavior of electroactive tissues [32,33]. Electroactive materials along with preparation of the proper substrate for cell adhesion and growth can make possible stimulation of cellular activity using electrical transfer [34,35]. In order to prepare proper environmental stimulus to develop healthy cell function and tissue regeneration, there is a need to develop scaffolds with all requirements, i.e., electrical, mechanical and chemical properties. The conductivity of tissues (ventricular muscle, nerve, lung, cardiac, and skeletal muscle) lies in an ordered manner in between 0.03 and 0.6 S/m [14,36].

**Figure 1:**
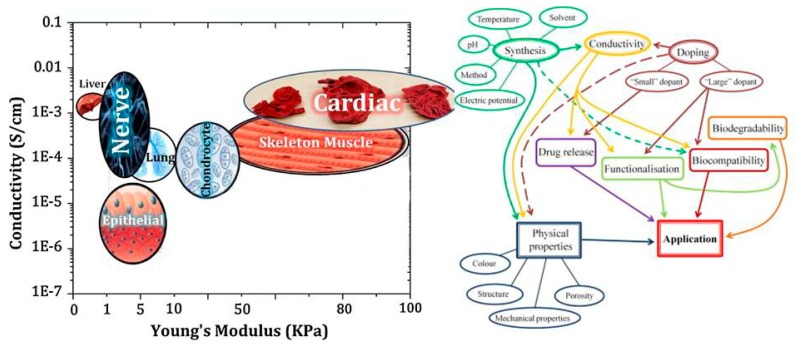
Conducive platform’s properties are adjustable with various tissues [14,43]. The plot in the left-hand side gives advice on selection of biomaterials for a target tissue considering their conductivity and mechanical properties, while the right-hand one provides the investigator with a brief view over microstructure–property–performance relationship when one takes first step in selection of conductive biomaterials for tissue engineering and regenerative medicine uses.

Conductive biomaterials are a member of a novel generation of “smart” biomaterials that let direct transference of electrical, electrochemical and electromechanical stimuli to cells. Electrically conductive organic polymers are a new type of ‘synthetic metals’ that merge the chemical and mechanical properties of polymers with the electronic confidants of metals and semiconductors, together [37,38]. These π-conjugated polymers have unconstrained electrons in their segments. Within the unsaturated segments, by free motion of the loosely held π-electrons an electrical path can be opened for itinerant charge carriers [32,39]. The CPs possessing useful electrical and optical properties effectively control the electrical motive, as well as have a high conductivity/weight ratio and can be manufactured with some key characteristics such as being biocompatible, biodegradable and porous. Moreover, the changes of surface zeta potential and polymer surface properties like wettability and spatial conformation can influence the cell’s behavior during ES [40]. Alternatively, cellular functions, such as cell growth, migration, adhesion, proliferation, and differentiation, can be corrected by conductive polymers with/without ES [41,42]. A great advantage of CPs is the ability to adapting their properties to the specific requirements of their usage by accommodating antibodies, enzymes and other biological segments [43]. As illustrated in Figure 1, conductive materials, due to versatility, can be designed for targeted tissue to enhance the regeneration. Substrate conductivity, which can be adjusted using synthesis method, can affect drug release pattern, physical properties, cell behavior and regeneration rate. Tissue properties can be encoded on the conductive substrate on which the designed platform recapitulates the tissue properties to achieve maximum regeneration.

**Figure 2:**
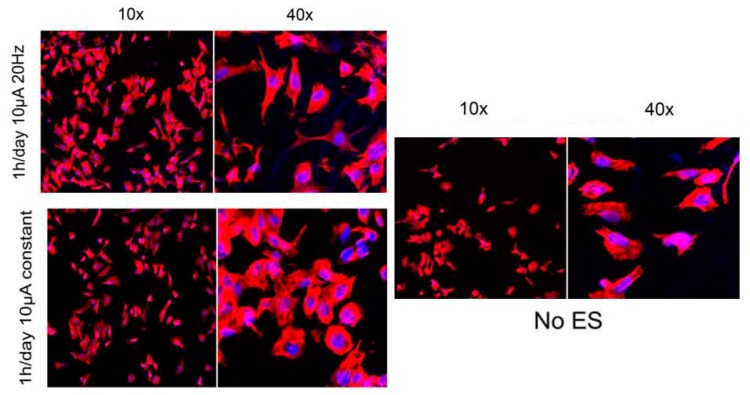
Effect of the electrical stimulation on the cell morphology and proliferation. Fluorescence microscopy of PC12 cells without stimulation, with constant 10 µA of stimulation and 10 µA, 20 Hz of stimulation. Electrical stimulation enhances cell proliferation. Amplitude stimulation affects the cell morphology [45], copyright Elsevier, 2011.

Langer and Ingber are the pioneers who verified the cell function interaction with CP by seeding mammalian cells on the conductive films based on PPy, affected by its redox state [39]. Later, Williams and Doherty exerted the PPy as a scaffold in nerve tissue engineering. Their results offered that this biocompatible conductive substrate could be used as a nerve conduit and as a substrate for electrical currents delivery simultaneously [44]. Furthermore, Thrikvikraman et al. showed the external electric stimulation of the stem cell can determine its fate to specific lineage. As illustrated in Figure 2, electrical stimulation and its response pattern have significant effect on cell morphology, proliferation and behavior. In this review article, conductive materials used to conduct the scaffolds for various tissue engineering applications have also been considered. Table 1 presents a brief view over conductive materials used in tissue scaffolds.

### 2.1. Polypyrrole

**Figure 3:**
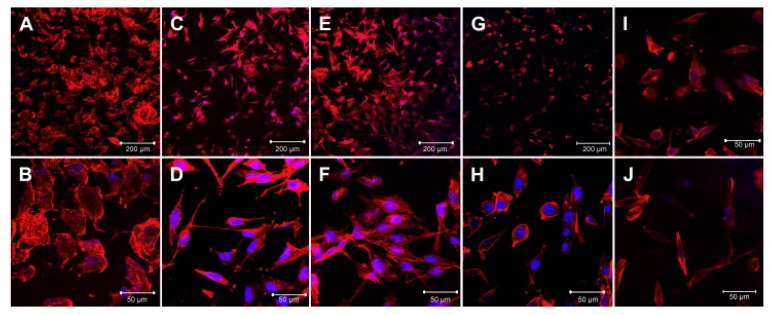
PC12 behavior on polypyrrole (PPy)/polycaprolactone (PCL) platform with various dopants. Cell proliferation on conductive polymer has been affected by dopant type which dramatically affect the cell proliferation, morphology and behavior. The different scaffolds are (**A**,**B**) PCL, (**C**,**D**) PCL/PPY-NSA, (**E**,**F**) PCL/PPY-DBSA, (**G**,**H**) PCL/PPY-DOSS, (**I**) PCL/PPY-PI, (**J**) PCL/PPY-lysine. Dodecylbenzene sulfonic acid (DBSA) and naphthalene sulfonic acid (NSA) as a dopant enhanced cell proliferation than others [50], copyright Elsevier, 2010.

Polypyrrole (PPy) is the most common choice among other conductive polymers due to high electrical conductivity, pliable procedure of preparation, various surface modification, great environmental resistance, proper biocompatibility, ion exchange capacity, and support cellular activities [48,49]. PPy should be doped with various anions such as Cl^−^, Br^−^, or NO^−^_3_ [48]. When opting to dopant it should be performed meticulously, for dopant affects the cell growth, proliferation and behavior. Runge et al. [50] As an example, the synthesized polycaprolactone (PCL)/PPY platform with various types of dopants and their effects are illustrated in Figure 3.

PPy is an attracting CP that has been frequently investigated for its efficacy towards the cell functions [46]. Since the nineties, PPy has been studied as a cell culture substrate within in vitro culture methods. In addition, animal models have been used to study the effects of implantation in vivo [33,49]. PPy have been utilized in artificial muscles, biosensors, drug delivery system, carrier of immobilized enzymes and tissue engineering [51,52]. For example, Bueno et al. electropolymerized PPy in xanthan hydrogels (XCA). Under stress XCA-PPy showed larger strain than the XCA, probably due to the slipping of planar PPy chains. Fibroblast proliferation was more enunciated onto XCA-PPy than onto XCA, due to its higher hydrophobicity and surface roughness [53]. Moreover, Haixia Liu and Ran Wan designed a biodegradable and electroactive scaffold consisting of magnesium (Mg), PPy-block-ploycaprolactone (PPy-PCL), and poly (lactic-co-glycolic acid) (PLGA) as a core-shell-frame mode for tissue engineering usage. Conductive PPy-PCL layer coated the Mg nanoparticles due to corrosion stability, through the UV-induced photo-polymerization procedure, and then PLGA is added to control the biodegradation of the illative composite. In vitro experiments using 293FT-GFP cells demonstrated that the scaffold was biocompatible [54]. Additionally, Guixin Shi and Mahmoud Rouabhia synthesized a new electrically conductive biodegradable material based on PPy nanoparticles and PLA using emulsion polymerization. Such substrate maintained a biologically significant DC current in a physiological milieu for 1000 h. Fibroblasts growth on such composite membranes was enhanced by the direct electron current applied through the membranes [52]. Deng et al. have synthesized cryogel based on PPY in which the platform exhibited the thermal sensitivity, shape memory and photothermal properties [55].

### 2.2. Polyaniline

**Figure 4:**
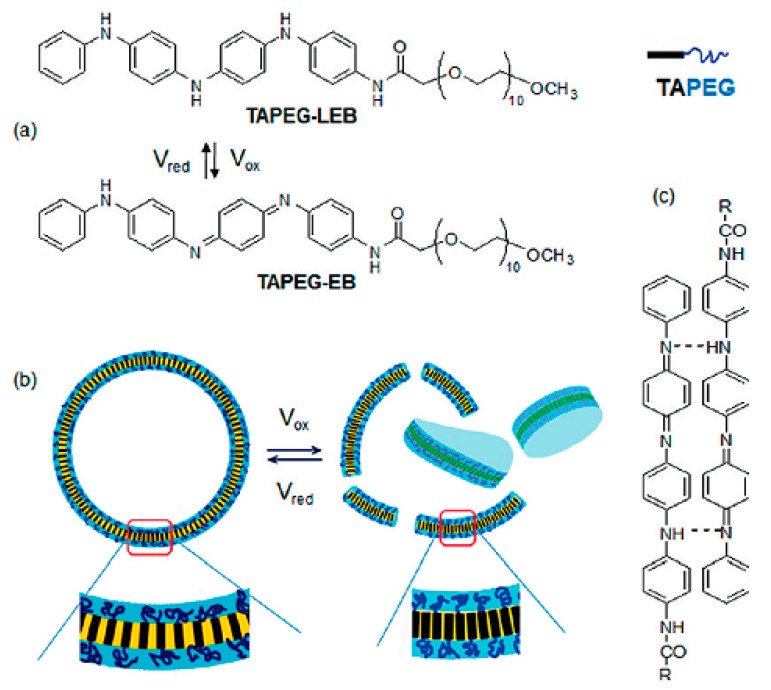
Electro-responsive tetraaaniline-PEG rod-coils in aquatic media, (**a**) chemical structures of TetraAaniline-PEG in the various oxidation states. (**b**) Redox switching results in vesicles rupture. (**c**) Hydrogen bonding of TAPEG [83], copyright American Chemical Society, 2011.

Polyaniline (PANi) is another best-determined CPs, which has variety of structural appearances, proper environmental durability and facility of charge transfer by the ‘doping/dedoping’ procedure [56,57,58,59]. In 1985, MacDiarmid et al. investigated PANi as an electrically reliable material [60,61,62,63]. Among CPs, PANi and its derivatives have achieved a growing portion of electroactive filed in tissue engineering. This was due to inimitable properties of this polymer including facile synthesis, variety of structural forms, superior thermal stability, high environmental constancy, in vitro compatibility, comfort accumulation of raw materials, and low cost. PANi has also been exhibited to have the potency to clean insidious free radicals from the environment, being a well troth to be used where tissues tolerate high oxidative stress especially post infarction [64,65,66,67]. Moreover, according to some reports PANi has proper antibacterial function, especially for Gram-positive bacterium [67,68,69]. Mattioli-Belmonte et al. illustrated and explained the biocompatibility of PANi in vitro and in vivo. A conductive form of PANi appeared when the nonconductive emeraldine base is doped with an acid [70]. PANi is the only CP whose electrical properties can be adjusted properly via charge-transfer doping and/or protonation, Furthermore, the amount of electrons associated with the polymer backbone does not alter during the procedure, being capable to be introduced as the only non-redox doping CP [71,72,73,74]. PANi has been determined to adjust cellular activities [75,76]. However, degradability is commonly a favorable characteristic in tissue engineering scaffolds, the key restriction factor is non-biodegradability of CPs, causing the inflammation and contributing to the further surgery for obviation [77]. To gadget this issue, supplying of low molecular weight oligoanilines (aniline trimer, tetramer and pentamer) has been examined by different researchers to display progressed processability and biodegradability [78]. Oligoanilines were observed to undertake conductivity versus high molecular weight analogous, while their low molecular weight nature assisted direct digestion by macrophages following kidney clearance. This, in turn, decreases the chance of adverse foreign body reactions. Therefore, representing these moieties into the backbone of intrinsically biodegradable material may represent a promising method for the manufacturing of conductive biodegradable scaffolds. It was speculated that the molecular weight, functional end group, and dopant exhibit strong effect on oligoaniline biocompatibility [79,80]. Self-dopant oligoaniline-based biomaterials can reduce the cell toxicity [81]. Numerous studies have considered this issue through blending oligoanilines with materials having biodegradable segments like ester linkages, fast degradable polymers like PLA, or combining with natural polymers. In fact, it has been proved that the electrical conductivity of the hyper branched biodegradable CPs is much higher than that of their linear counterpoint with similar amounts of aniline pentamer [82]. Aniline oligomer-based biomaterials have been used as an on-demand electrical drug release. In aqueous media, aniline oligomers tend to self-assemble and form vesicle and particle, which can encapsulate loaded drugs in scaffold. Applying electrical current can rupture the vesicle and release the drug (Figure 4) [83]. 

### 2.3. Poly (3, 4-ethylenedioxythiophene)

Poly (3, 4-ethylenedioxythiophene) (PEDOT) has lately been prospected as an option to PPy since it has higher resistance to oxidation and more conductivity. Contrary to PPy, PEDOT can preserve 89% of its conductivity under same conditions [84]. Its high surface area and unique structures resulted in lower impedance which enhance its usage in bioelectrode coatings. In vitro toxicity and biocompatibility tests have shown that PEDOT, like PPy, has no cytotoxic effects on cells [85]. Xufeng Niu and Mahmoud Rouabhia showed that a sorely thin coating of PEDOT on micro fibrous PLA network is of adequate conductivity and durability in hydrous milieu to maintain ES to cultured fibroblasts. PEDOT-coated fibers exhibited higher hydrophilicity, thermal stability, and lower glass transition temperature contrasted with the pristine PLA fiber. In a cytotoxicity test result, PLLA/PEDOT scaffold showed no cytotoxicity and supported human dermal fibroblast migration, adhesion, and proliferation [86]. PEDOT was utilized for neural recording applications which in comparison with gold exhibit low impedance and high charge density; moreover nanofiber structure augmented its performance [87,88].

### 2.4. Polythiophene

The polythiophenes (PTh) are a group of CPs, which chemical modifications manifold their properties to suit various applications. The advantages of PThs over other conductive monomers (such as PPy) are that they are bowed to functionalization using a wide range of reaction conditions [89], which can be used in doped and neutral states with various properties [90]. Thiophene, terthiophene, and bithiophene molecules as parent monomers are used to synthesize the polythiphene derivatives with different functionality and tailorable properties for various applications [91]. Equated to investigations on PPy and PANi, studies on the suitability of PTh and its formatives in tissue engineering are fewer, and proportionately recent. The PThs were showed to have properties similar to, and in some cases more convenient, than other CPs [92]. 

### 2.5. Carbon

Naturall-y-occurring carbon allotropes come from variations in covalent binds of carbon atoms. Each of the carbon allotropes has specific properties based on the unparalleled spatial regulation of carbon atoms. Carbon allotropes comprise fullerene, diamond, DLC, graphite, and carbon nanotubes (CNT) [47,93,94]. “Graphene”, referring to the isolated two-dimensional crystal structures made of single atomic layers of graphite, presents great thermal and electrical conductivity due to its inimitable structure and strong carbon–carbon bonding. Furthermore, low defect density in the crystal lattice provides superior thermal and electrical conductivity in single layer graphene. Excellent electrical conductivity and thermal properties of graphene can be beneficial not only in electrical instruments but also in biomedical devices in order to measure cell potential and as a conductive platform in tissue engineering and biosensors [95,96,97,98]. It has been shown that accompanying graphene to polymers can elevate the electrical, mechanical, and thermal properties of the originating nanocomposites [99]. Recently, researches have shown that graphene membranes and hydrogels with high in-plane stiffness, can potentially be used as a biocompatible and transferable substrate for stem cell culture [100,101]. CNTs can be assumed to form when a graphene sheet is twisted into a cylinder. Single walled carbon nanotubes (SWNT) form when one graphene sheet is rolled up, while extra concentric graphene sheets create a multiple wall carbon nanotube (MWNT). In addition, carbon nanofibers (CNFs) with poor arrangement of atoms form when graphene sheets are bent at some angle to form embankment of nanocones [102,103,104,105]. CNTs are hollow nanostructures containing carbon atoms bound to each other using sp2 bonds, of which the key roles are bringing CNTs’ high electrical/thermal conductivity and mechanical properties. Excellent electrical conductivity of this aromatic structure is due to the fourth valence electron which is shared and mobile [106]. In 1991, Lijima discovered CNTs for the first time [107]. Since then, CNTs and CNFs have possessed increasing consideration due to their thermal, mechanical, optical, electrical, and structural features [102,108,109]. Carbon meshes used in tissue engineering have frequently been provided from CNFs and CNTs, either single wall or multiwall and graphene [110,111,112]. Their inimitable electrical and mechanical properties can be handled to form biomimetic tailored scaffolds [113]. Some arguments exist in concerns to the biocompatibility of SWNT and MWNT, with some in vitro studies demonstrating that CNT has a cytotoxic effect while other studies reporting CNT to be an optimum platform for cellular growth [110,111,112]. Functionalization of CNTs with active molecules makes them biocompatible which can be utilized in biomedical applications [114]. Rare attempts have been carried out to expand pristine CNT-based substrate with lack of mechanical properties and elevated CNT toxicity, where the concatenation of CNTs within polymer composites has appeared as an arresting mostly invested [115]. For example, in 2008 Sanjib Bhattacharyya and Samuel Guillot designed a nanocomposite consist of CNTs, by dispersing functionalized SWNTs in hyaluronic acid (HA) solutions in order to form hybrid HA hydrogels with SWNTs cross-linking using divinyl sulfone. This resulted in a significant variation in the topology of the hydrogels. The authors observed that the incorporation of 2% wt SWNT (vs HA) does not change water uptake capacity, while dramatically altering the dynamic mechanical properties of the hybrid hydrogels versus pristine ones [96]. Furthermore, Hermant et al. reported the development of two species of SWNTs, namely, HiPCO SWNTs and Carbolex SWNTs in a SWNTPS/PEDOT: PSS system. The authors determined that in the composite with PEDOT: PSS the SWNTs do not mostly chip in the conductivity and reasonably act as a scaffold that assists the formation of an electrically infiltrating network of the PEDOT:PSS [116].

### 2.6. Silicon

The amorphous SiOx cover on single crystalline silicon core enables several modifications on Silicon nanowires (SiNWs) which are pertaining for biosensor and tissue engineering applications [117]. SiNWs have tunable electrical conductivity, harmonic dimensions, and comfortable surface adjustability [118,119]. Additionally, many researches proved the SiNWs’ biodegradablity, and that Si(OH)_4_, their main degradation residues, are metabolically enduring in vivo. This makes them profitable compared to other non-biodegradable, electrically conductive nanomaterials, particularly for in vivo applications’ capability [119]. Nanostructured silicon as a biomaterial application has been largely reinforced by reports of easily calcium phosphate growth on the Si surface as an important bioactive material [120]. 

### 2.7. Gold

Gold nanoparticles (AuNPs) are achieving considerable attention due to their biocompatibility and relative ease of functionalization with various organic and biological moieties [121,122]. AuNPs have been successfully applied in many biological implementations to make a inimitable cellular environment that merges controllable conductivity and elasticity, which are favorable for tissue engineering [123]. McKeon-Fischer and Freeman demonstrated that low cell proliferation is not due to Au toxicity but may be a sign for myotube differentiation. An electroactive, biocompatible, and biodegradable scaffold containing electrospun PLLA nanofibers with high amounts of AuNps, was prepared for skeletal muscle tissue engineering that could perhaps need lower voltages to develop myotube formation [124]. Despite the fact that AuNps are settled of an inactive material, biocompatibility distributions have to be taken into account. Modification with AuNP alters material nanotopography which could affect a vast range of cell activities. Cells in contact to AuNps go under a phagocytosis process and therefore the particles are accumulated inside the cells in perinuclear sections (structures adjacent to the cell nucleus). Regarding cytotoxic effects, one has to recognize the effects related to the nature of the material as well as the effects prevalent to nanoparticles. At the same time, inert particles such as gold expressed tissue inflammations due to the presence of gold nanoparticles within the cell [125]. However, in cell culture experiments AuNps are considered as biocompatible, and serious cytotoxicity has not been observed yet [125,126], as well as in in vivo and pre-clinical scenarios [127,128,129,130].

### 2.8. Melanin

Melanin is a light absorber polymer achieved from the oxidation of tyrosine. Melanin is widely interspersed in animals and plants. It is the main pigment existing in the vertebrate’s surface [131]. Eumelanins is the main component of melanin, in which it could be the extended heteropolymers of 5, 6-dihydroxyindole and 5, 6dihydroxyindole-2-carboxylic acid. These heteropolymers can gather to form accumulates with strong π-π interactions, which supply the chemical foundation for the inimitable features of melanins.

Although the exact conduction mechanisms are not clear, physical form, temperature, and the hydration state are three factors that affect the electrical conductance of melanins, which largely ranges from 10^−8^ S/m to 10^−3^ S/m. The inimitable electrical properties of melanins propose their potential application as conductive biomaterial scaffolds in cardiac tissue engineering [132,133]. Melanin is a natural photoprotectant of skin and hair with antioxidant features, which can be used in Parkinson’s disease [134]. For instant, Dan Kai et al. suggested conductive nanofibers including 10% melanin that ameliorates cell interaction. Furthermore, improved electrical stimuli transfer (rectangular, 150 ms, 1 V/cm, 1 Hz) through the scaffolds exhibited improved cell proliferation, alignment, coupling, and the expression of connexin-43 [132].

### 2.9. Calcium Titanate

Calcium titanate (CaTiO_3_) possesses an orthorhombic architecture at ambient temperature, and the structure turns into tetragonal at 600 °C and cubic at 1000 °C. From the optical density studies on single-crystal CaTiO_3_, Linz and Herrington determined the band-gap energy of 3.4 eV (at 300 °K) [135,136]. Thrivikraman and Mallik used spark plasma sintered HA-CaTiO_3_ as a pattern system to determine the influence of altering conductivity on cell behaviors. In addition, mouse myoblast cells (C2C12) were seeded on scaffold which observed that the cell proliferation was enhanced. Generally, this work convincingly appoints the favorable effect of the platform conductivity to cell proliferation and differentiation besides corroborates the efficiency of HA-CaTiO_3_ biocomposites as conductive substrate to comfort the growth and proliferation of myoblasts, even when seeded without exterior electric field [137].

## 3. Application of Conductive Materials in Tissue Engineering

### 3.1. Nerve Tissue Engineering

In advanced community, recuperation from spinal cord injuries (SCI) and neurodegenerative diseases (NDD) enumerates one of the biggest universal general health challenges [138]. For in vitro/vivo studies, the principal demands for the substrate are non-cytotoxicity and mechanical properties of scaffolds necessitate to be appropriate for neural tissue expansion. Moreover, surface topography and intrinsic porosity of the scaffold affect cell proliferation and differentiation. Human body reacts to electrical stimulations and the key incorporator of neural transmission in the body is the action potential created at the synapse. This infers that a perfect neural scaffold should also own electrical conductivity to assist neurite outgrowth and thereby elevate nerve regeneration in culture [139].

Neurons have a potential of using comparatively weak electrochemical currents in mV range for controlling cellular activities. Electrically-conductive substrates can help to transfer these necessary signals among neurons, which have a positive effect on the expansion of neural tissue. Conductive scaffolds were used in nerve tissue engineering. Pires et al. cross-linked PEDOT: PSS, then neural stem cells were cultured in laminin coated substrate, and differentiated over eight days in the lack of those factors under 100 Hz pulsed DC electrical stimulation, 1 V with 10 ms pulses. The total number of neurons was 1.6 times higher with longer neurite for cells cultured under electrical motivation. Such stimulations were also directed to longer neurons. It was the first time that PEDOT:PSS combination was used to extend human neural stem cells through the implementation of pulsed signals, influencing on their differentiation directed to neurons and promoting to longer neurite [140]. Huang et al. [141] fabricated a biodegradable conductive composite containing PPy and chitosan for electrically stimulation of Schwann cells. Their results revealed that low potential (100 mV/mm) stimulations can have useful effects on cellular activities but superior potentials (300–1000 mV/mm) have damaging influences. Neurite outgrowth was highly elevated by electrical stimulation when electrical stimulation was applied through the conductive scaffold in vivo. Altogether, Schwann cell production of nerve growth factor (NGF) and Brain-derived neurotrophic factor (BDNF) was considerably elevated by electrical stimulation, which might further contribute neurite outgrowth and nerve regeneracy [142].

Thitima et al. constructed a new biomaterial for neural tissue engineering applications by coating electrospun PLA nanofibers with an electroactive polymer, PPy, via admicellar polymerization. Cell culture experiments demonstrated that PPy-coated electrospun PLA scaffold has no toxicity in vitro and could promote adhesion and immigration of neural progenitor cells. It should be noted that the PPy-coated random fibers were accidently oriented and had innumerable connections between coated fibers, while the organized fibers gave more electron current path along the PPy surface [143,144]. One of the main hindrances involving the field of electrically CPs such as PANi is difficult processing and therefore electrospinning of PANi still continues a great problem. To solve this challenge, most of the researchers have electrospun PANi by blending it with other spinnable polymers, though it reduced the conductivity of the composite fibers. For example, Prabhakaran et al. in their study designed electrospun conductive nanofibers of PANi/PLLA. Electrical stimulation along this conductive nanofibrous scaffolds showed elevated cell proliferation and neurite outgrowth versus the PANi/PLLA scaffolds that were not subjected to electrical stimulation [21]. As it was discussed recently, PANi is prevalently being used for the preparation of scaffolds which can electrically motivate cells so that regulate specific cellular functions and, eventually, the procedure of tissue regeneration.

**Figure 5:**
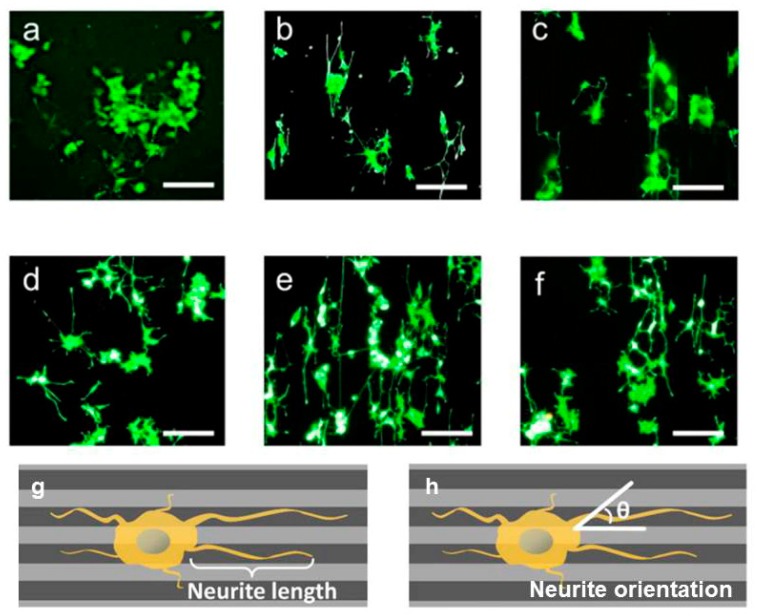
Culturing Schwann cell on conductive substrate affected the cell morphology and orientation. Moreover, micropatterning along with conductivity affect the cell morphology and alignment. (**a**) flat substrate, (**b**) grooved substrate 50 μm, (**c**) grooved substrate 100 μm, (**d**) flat conductive substrate, (**e**) grooved conductive substrate 50 μm, (**f**) grooved conductive substrate 100 μm, (**g**) schematic of neurite length, (**h**) neurite orientation [145], copyright Elsevier, 2018.

Engineered scaffold can affect the cell alignment and elongation. Micropatterned conductive substrate based on poly (glycerol sebacate) and aniline pentamer was synthesized and Schwann cell and PC12 were seeded on. It was observed that, such platform along with enhancement of the cells alignment and neurite elongation increases the nerve growth factor (NGF) gene expression of Schwann cells. Comparison between Figure 5a–c and Figure 5d–f, it divulged that seeded cells on conductive substrate exhibit the multiple neurite from cell bodies, more neurite terminus and longer neurite length [145]. 

**Figure 6:**
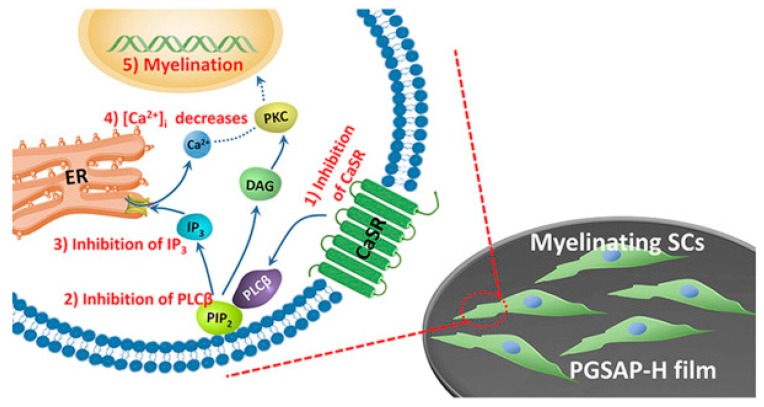
Mechanism of Schwann cells’ (SCs’) myelination on conductive platform. Conductive films inhibit CaSR and PLCβ pathway, and then decline the intracellular Ca2 þ level [25], copyright Elsevier, 2016.

Guarino et al. synthesized PANi and hydrogel cross-linked incorporated to allow the preparation of materials with good conductive manner. The attendance of PANi obviously picked up the conductivity of the material to (1.1 ± 0.5) × 10^−3^ mS/cm with a PANi content of 3% wt. In vitro studies corroborated that 3% wt PANi also promotes the biological reaction of PC12 and hMSC cells. PANi/Polyethyleneglycol diacrylate macroporous hydrogel add new usefulness regarding morphological and conductive attributes, both of which are necessary requirement to guide neural cells in regenerative pathways [146]. Wu et al. synthesized the conductive polyurethane based on aniline oligomer which ameliorated the Schwann cells myelin gene expression. It was speculated that the CaSR and PLCβ pathway was blocked by conductive substrate and then Ca^2+^ level of intracellular decreased. Intracellular Ca^2+^ level and DAG decrement resulted in PKC inactivation; hence, protein kinase enzyme activated other signal pathways like mitogen-activated protein kinase (MAPK) which can affect the cell behavior like differentiation, migration and secretion (Figure 6) [25].

Zarrintaj et al. synthesized the polysaccharide based conductive substrate with various length of aniline oligomer [147,148,149,150]. It was reported that the conductive polysaccharide can be good candidate for neural regeneration because of resembling ECM [151,152,153].

Conductive platform along with directs electrical signal transfer, assists neural growth. Functionalization and bioactive coating are suggested to ameliorate the biocompatibility of CNT-based scaffolds. According to Mattson et al. aldehyde 4-hydroxynonenal is desired because of its influences in raising intracellular Ca^2+^ concentration, improving cytoskeletal proteins and signaling pathways that direct neurite expansion [154]. Using this plan, MWNTs were coated with 4-HNE to control neural cell activities. Results demonstrated that SWNTs can be functionalized to promote neuron growth and expanding that is decisive in neural regeneration [102]. Moreover, Kabiri et al. compared the neural differentiation and proliferation of stem cells on three distinctive aligned electrospun scaffolds composed of PLLA complemented with either SWNT or MWNT. Adding nanotube impregnated conductivity to the scaffolds and directed mouse embryonic stem cells for neural differentiation as proved by a development of mature neuronal markers expression [155]. 

The neural stem cells as a multipotent cell type in the CNS, which display undertaking outlooks in expansion cell therapies for neural regeneration. A scaffold that adjusts neural stem cell action and tissue improvement by constructing milieu has been a perfume in clinical usages. At the present time, the combining carbon nanomaterials propose numerous occasions to prepare novel scaffolds as neural tissue engineering. Li et al. [156] revealed that the application of graphene foam as a novel platform for neural stem cells in vitro. A good electrical connecting of 3D-GFs with differentiated NSCs for impressive electrical stimulation was seen. Their research signified 3D-GFs could tender a formidable intent for neural stem cell research, nerve tissue engineering, and neural interface. The cyclic voltammetry results implied that it involves low risk for three-dimensional graphene foams (3D-GF) to electrically stimulate cells via capacitive charge injection like 2D graphene electrode. However, due to its high surface area, it could supply much stronger charge injection capability than 2D graphene films with the same geometrical area. As a result, the 3D architecture of GFs can impressively enhance the electrical stimulation actions of conductive scaffold. Furthermore, it was found that in contrast to two-dimensional graphene films, 3D-GFs can promote neural stem cells growth and Ki67 expression [30]. Table 2 summarizes the conductive scaffold for neural regeneration.

### 3.2. Cardiovascular Tissue Engineering

Cardiovascular disease is the most prevalent reasons of death in developed countries and it is becoming an epizootic menace of the 21st century [188,189]. Among different strategies, tissue engineering and cell biology have recently found some innovative ways to shed light over finding new treatment approaches. It is believed that the exact function of cardiomyocytes (CM: contractile muscle cells that are specialized for myocardium tissue) and neurons is supported by on persevering conductivity. It is also known that heart disease or improper functioning may interrupt such conductivity [190,191]. Myocardial infarction (MI) is the most usual reason of heart failure and distraction [123,192,193]. Behind an immense CM loss owing to MI, the myocardial tissue misses considerable intrinsic regenerative ability to swap the lost cells, thus the disorder of the heart wall muscle is everlasting [194,195].

**Figure 7:**
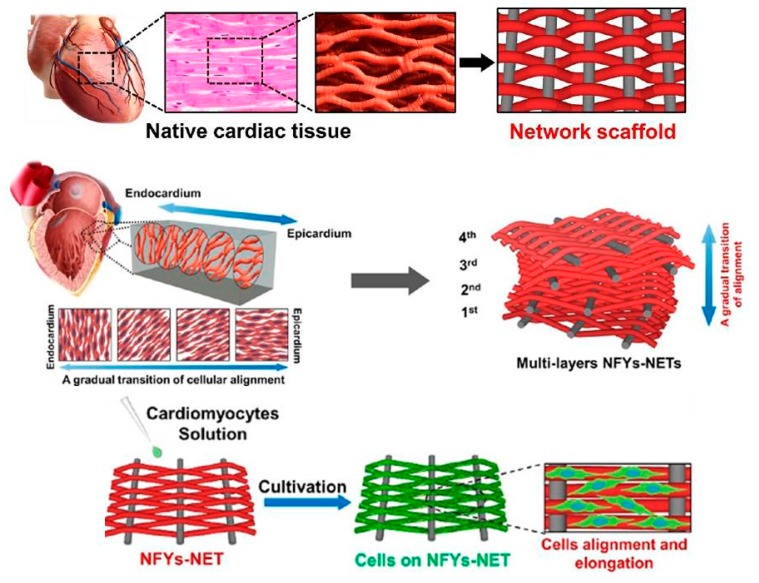
An interwoven structure of cardiac with aligned cell layers and biomimic scaffold with similar structure. Multiple layers of Yarn nanofiber can recapitulate the cardiac muscles which cause cell alignment and elongation [200], copyright American Chemical Society 2017.

Numerous methods have been used to enhance CM and neuron development near defunct tissue after a MI. Such projects possess ex vivo culture of CM on cardiac patches for direct cell injection, probable implantation, scaffolds based on collagen, PLA, PCL, 3D printing, and injectable scaffolds using materials adorning from fibrin to CNFs. Each method has its own superiorities however usually all of the above can be detached into two categories: (a) Conductive patches and (b) non-conductive patches [196]. One of the most trustworthy tactics among the above is cardiac tissue engineering [197]. The major difficulty in tissue engineering is to imitate the structural and functional properties of ECM, thus constructing a bioactive platform possess suitable chemical, biological, and conductive properties [198]. Versus the natural heart tissue milieus, the porous substrate have a few artificial characteristics. First, the scaffolds are non-conductive at biological frequencies, but heart tissues have a DC conductivity of about 0.1 S/m and are emphasizing with electrically conductive Purkinje fibers. Secondly, many scaffolds do not have nanofibrous structures at nano scale round 10–100 nm diameters, which are plentiful in natural ECMs and possess a crucial role in controlling cellular functions. Thirdly, the scaffolds have typically more weak mechanical properties than the native heart muscles [199]. Wu et el. synthesized the interwoven directed conductive nanofiber to recapitulate the cardiac tissue. Figure 7 illustrates the cardiac tissue structure which was the inspiration of 3D scaffold fabrication. 3D yarn/hydrogel scaffold mimicked the cardiac structure and conducting 3D cellular arrangement. Such scaffold enhanced the cellular orientation and proliferation which can be a harbinger for ultimate cardiac regeneration [200].

**Figure 8:**
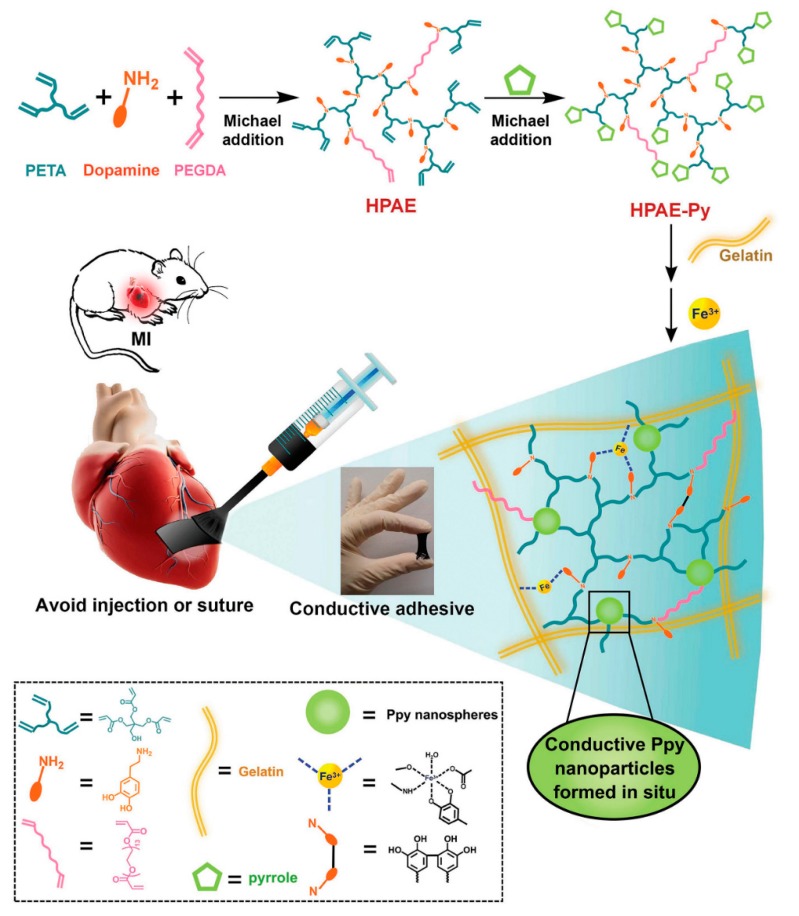
Conductive adhesive hydrogel patches synthesis attached on myocardial infarction (MI) site. Pyrrole was capped dopamine, simultaneously polymerized using Fe^3+^ oxidation which acted as an adhesive and conductive substrate [202], copyright John Wiley and Sons, 2018.

Electroconductive materials have been acknowledged to be useful for myocardial tissue engineering due to the capability to be used as a substrate that controls electrical stimulation [132]. Li and colleagues showed that nanofibrous scaffolds made of gelatin and PANi as a conductive platform supported H9C2 annex and proliferation. This study demonstrates the first phase in their long-term tactics of seeding cells on nano-fibrous scaffolds made of CP. Conditional upon the concentrations of PANi, the cells primarily showed different topologies on the fibrous scaffold [38]. Later Dan Kai and coworkers reported an electrospun PCL-gelatin-PPy scaffold that advanced CM annex, proliferation and presentation of cardiac-specific proteins. Nanofibers made of 15% PPy represented the most equivalent properties of conductivity, mechanical properties, and biodegradation, corresponding to the provisions for repairing of cardiac tissue [192]. After that Benjamin Spearman et al. demonstrated that after culturing CM on the conductive PPy-PCL substrates, more cells were distinguished to possess peripheral localization of the gap junction protein connexin-43 [201]. This composite had a resistance of 1.0 ± 0.4 kΩ cm, which is equivalent to natural cardiac muscle. Consequentially, faster calcium wave spreading rate and shorter calcium temporary period for CM monolayers on PPy-PCL associated to cells on PCL [201]. Liang et al. synthesized the cardiac patch based on PPy-dopamin which was applied using injection (Figure 8). Such method attracted a significant attention because of its non-invasive nature and elimination of surgical operation. After injection, hydrogel formed and due to its conductivity, which is equal to normal myocardium is powerfully bonded to the beating heart. It is a promising method to eliminate the surgical operation [202]. 

The cooperation between conductive scaffold and stem cells offers a chance to prevail MI. Borriello et al. determined that the conducting PCL/PANi platform could be done as a suitable substrate to promote MSC differentiation to CM lineage. Their results proposed that PANi short fibers conduct electrical stimulations impressively. The authors revealed that the viability of CM-like cells onto PCL/PANi substance was considerably superior to that on the PCL. Furthermore, the presentation of sarcomeric α-actinin was also approved on a conductive platform [203]. Moreover, Spencer W Crowder and colleagues introduces an electrospun PCL including CNT to specify MSCs fate [204]. This scaffold surrounded inherent potency enhancing rod-like and prolonged morphology in 3D culture. Outcomes showed that differentiation of hMSC can be improved by culturing on conductive scaffolds [204]. 

Additionally, the exhorting actions gained by using CPs, for instance, CNTs with nanostructure can emulate the ECM. Valentina Martinelli and colleagues showed that CNT platforms assist CM growth and puberty by changing the gene expression program, doing the cell electrophysiological attributes and enhancing networking and puberty of syncytia. They showed that ventricular myocytes cultured on scaffolds of MWNT communicate with CNTs by forming firm osculations and show enhanced survival rate and proliferation [205,206]. Later Xia Li et al. indicated that the poly (N-isopropylacrylamide) (PNIPAAm)/SWNTs hydrogel showed considerably higher bioactivities to encapsulated stem cells compared to one-fold PNIPAAm hydrogel, containing improving cell attachment and proliferation, in vitro. Furthermore, when acting as a vehicle for intramyocardial delivery of stem cells after MI, the PNIPAAm/SWNTs gel considerably assisted the hybridization of culturing cells in infarct myocardium and increased their therapeutic efficacies [207]. Lately, Stout et al. [190] presented a PLGA-CNF composite for cardiac tissue engineering. Aortic endothelial, fibroblast, and CM cells were seeded onto a PLGA: CNF scaffold to distinguish if CNF concentration has an influence on cellular activity. Throughout consecutive ES, CM cell density increased compared to its static counterpoint. A lesser raise in Troponin I excretion in ES in comparison to the static state demonstrated nominal CM cell activity within cell cultures. Fibroblast and endothelial cell growth studies demonstrated the material prevented or stopped proliferation during both static and ES, thus promoting the growth of CM onto the damaged tissue area. Moreover, the findings showed that CNF concentration exhibited an influence on PLGA:CNF scaffold in vitro compatibility features with the proper results achieving from the 50:50 (PLGA:CNF) [208]. Furthermore Kharaziha et al. fabricated hard and flexible hybrid scaffolds with improved electrical attributes containing CNTs detruded aligned poly (glycerol sebacate): Gelatin nanofibers [196]. The resulting CNT-PG scaffolds showed more powerful instinctive and simultaneous beating action evaluated to those seeded on PG scaffold. Totally, their results showed that aligned CNT-PG scaffold have excellent mechanical attributes with elevated CM beating attributes [196]. 

Dissident to the usefulness of cell-based therapies in regenerating infarcted myocardial tissues, lack of electrical communications between donor cells and the host myocardium is caused by the absence of functional merging of them. To reconstruct the contractile heart muscle, electroactive scaffolds are employed in order to graft implanted cells with the host tissue in a simultaneous strategy. The electroactive portion of scaffolds signifies identical stimulatory of implanted and host cells, and also promote distribution of the electrical wave front [119,209]. Chun-Wen Hsiao et al. suggested a nanofiber mesh of PANi and PLGA, as a conductive layer for adapting the beatings of the seeded CMs simultaneously [209]. Accordingly, after electrical stimulation contractions of the single clusters grown on the conductive platform were contemporized, and the cell beating frequency was similar to the electrical potential [209]. Su Ryon Shin and coworkers specified that the presence of CNT as a conductive factor in gelatin-methacrylate hydrogel resulted in promoting myocardial cell attachment, organization, and cell–cell communication [199]. Moreover, 2D engineered cardiac tissue on CNT-gelatin-methacrylate showed suitable mechanical unity tolerating tissue constriction. Combination CNT to the structure decreased stimulation limen about 85% lower. Actually, CNT networks connected the insulating pore walls of the hydrogel, supplied additional paths for straight electrical charge flow, and decreased the impedance between cells for charge redistribution and action potential dissemination [199]. Furthermore, Hongyu Sun et al. introduced SWNTs blended into collagen scaffolds were used as growth carriers for CMs, which promoted CM adhesion and proliferation [210]. Moreover, they found that the presence of CNTs significantly incremented intercalated disc (ID: a well-organized structure that attaches myocytes into a syncitium electromechanically and supply propagation of electrical diagonals in every part of the heart) protein expression and improved ID congregation and activity. On that foundation, they further probed the fundamental mechanism for how CNTs promoted ID assembly. They discovered that the signaling pathway of β1-integrin-mediated intervened CNT-induced regulation of mechanical and electrical junction proteins. Particularly, CNTs significantly precipitated gap junction formation using β1- integrin-mediated FAK/ERK/GATA4 pathway activation. These results supply a noteworthy understanding into the mechanistic influences that CNTs cause on neonatal CM actions [210].

Electrical signals’ dissemination through natural myocardium causes ordered constriction of the heart, then blood pumping throughout the body. An unfit mechanical junction between CMs terminates to an annihilated cardiac pumping; presented improper electrical junction may end to incomplete conduction of the electrical impulse and pursuant pickup of cardiac arrhythmias. Tal Dvir et al. declared that flexible gold nanostructure in alginate hydrogel can merge the insulated pore walls of alginate and promote electrical connection between cardiac cells and their neighbors. In this way, thicker- and further-aligned tissue grown on this platform and cells in these tissues contracted simultaneously [211]. Elsewhere, Tan et al. specified that adhesion of a trace amount of conductive silicon wires in differently scaffold-free cardiac spheroids can create an electroactive mesh, outstanding to simultaneous and considerably improved constriction, terminating in more developed contractile maturation and cellular structural [119].

The study mentioned earlier is the first explanation of using nanostructure semiconductors to advance cardiac tissue formation and CM maturation without importing contractual scaffolding materials [119]. Notwithstanding all these attempts, it is regarded that the usage of electroactive biomaterials is just restricted to in vitro maturing engineered cardiac tissues, and it subsists to be elucidated whether electroactive biomaterials can advocate functional engineered cardiac tissue-formation in vivo, perform beneficial traces on the heart function, or claim the structural and functional accretion between engineered cardiac tissues and infarcted myocardium based on their nanoscale attributes [204,212]. One challenge is weak cellular totality in engineered cardiac tissues constructed using current procedures. This is particularly reflected in the insufficient regenerating of IDs [210,211,213]. 

Arterial blood vessels have a multi-layer construction including collagen and elastin fibers, smooth muscle, and a complex construction of endothelium. Blood vessels have a different round structure due to the orientation of their fibrous parts [214]. A.S. Rowlands for the first time showed that vascular smooth muscle cells (VSMCs) seeded on a CP layer and subject to ES not only present improved proliferation but can be contemporaneously patronized to excess contractile protein expression [168]. In this research VSMCs were seeded on PPy layer and were exposed to a 50-mA sinusoidal ES at 0.05, 5, and 500 Hz. Such layers were coated with collagen IV followed by Matrigel and doped with HA in order to imitate the tissue and promote cell adhesion. Enhanced proliferation and expression of smooth muscle phenotype markers (smooth muscle α-actin and smooth muscle myosin heavy chain) were monitored in cultures stimulated at 5 and 500 Hz [215]. Moreover, Mihardja et al. prepared an injectable hydrogel of alginate merged to PPy as a conductive platform. Local injection of polymer mixture in to the infarct area produced considerably better levels of arteriogenesis. Moreover, this scaffold considerably improved migration of myofibroblasts into the infarct zone [216]. The conductive scaffold for cardiovascular regeneration is summarized in Table 3.

### 3.3. Bone Tissue Engineering

**Figure 9:**
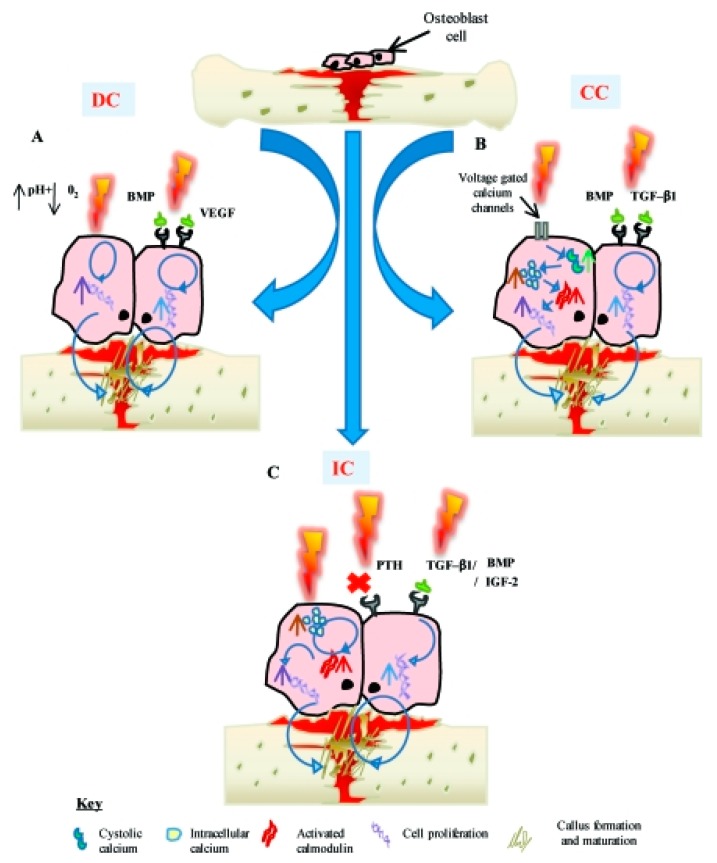
(**A**) Direct current decreases the oxygen level and enhances the pH, which causes to increase of osteoblast proliferation (**B**) capacitive coupling results in increment of cystolic calcium through voltage gated calcium channels. (**C**) Inductive coupling stimulation results in a direct enhancement in intracellular calcium [235].

In 1950, bone was found to present inherent electrical attributes such as piezoelectricity [229]. These attributes create an internal electrical field in response to tensions that change cell proliferation, which can describe why exterior electric and electromagnetic excitation have an advanced effect in bone healing therapy [230,231,232,233]. The electrical potentials happened in bone due to the mechanical loading, which is described in terms of both the piezoelectric features of the collagen in bone and by the motion of ionic fluids through the structure. These potentials have been connected to the mechanical conformity of bone in response to loading, outstanding to the recommendation that using an electrically active part in an implant material may enhance healing and conformity of the circumambient tissue [234]. It was shown that such excitation improve osteoblasts activity. Figure 9 exhibits various electrical stimulation methods which have been utilized for bone regeneration. DC decreases the level of oxygen and enhances the pH; hence, osteoblast cell proliferation increases. In second method, capacitive coupling results in an increment in cystolic calcium through voltage gated calcium channels and finally inductive coupling result in a direct increment in intracellular calcium, which increases activated calmodulin stores [235]. 

Progress studies in 3D scaffolds prepared for bone tissue engineering are frequently afforded to enhance the attributes of the scaffolds in regard to their chemical and mechanical attributes. To incorporate the tissue engineering methods with the idea of improving the bone healing by electrical stimulation, the electrical attributes of the scaffolds needs to be corrected [230]. The CP such as PPy and PANi have been vastly used and researched due to their comfortable and helpful synthesis, manageable electrochemical activity, and great compatibility in vitro. Haitao Cui et al. in their research prepared a novel electroactive polyelectrolyte based on tetreaniline and poly (L-glutamic acid), that manufactured through layer-by-layer (LbL) assembly strategy. In comparison to the nongrafted polyelectrolyte multilayer films, the tetreaniline-grafted samples demonstrated superior stiffness and roughness in micro/nano structures. The surface specifications and the typical electroconductive attributes were more useful for cellular activity of preosteoblasts MC3T3-E1 cells. Furthermore, the improved influences were seen on the incorporation of MC3T3-E1 cells, when the electroactive polyelectrolyte multilayer films were coupled with ES, particularly in the initial phase of the osteoblasts differentiation [236].

Since the 1980s, CPs with admissible biocompatibility have been applied in several biomedical usages. CPs intervene ES and have the capability to be the motivating factor that increases bone regeneration. PEDOT is a biocompatible CP which is lately being operated in biomedical usages particularly in bone tissue engineering. Shahini et al. in their research fabricated 3D conductive scaffolds by hiring a biocompatible CP, i.e., PEDOT:PSS in the optimized nanocomposite of gelatin and bioactive glass. Adult human MSCs were cultured on the substrates, for in vitro examination. Their outcomes demonstrated that such conductive scaffolds are not only structurally desirable for bone tissue engineering, but also can be an approach in incorporating the tissue engineering methods with the method of improving the bone healing by electrical stimulation [237]. In 2014, Liu and Cui showed the cytocompatibility of the aniline pentamer-graft-gelatin/PLLA nanofibers in vitro by the attachment and proliferation of MC3T3-E1 cells. The cellular expansion was considerably larger on electroactive aniline pentamer-graft-gelatin/PLLA nanofibers than on PLLA nanofibers. Moreover, the aniline pentamer–graft–gelatin/PLLA nanofibers motivated by an electrical pulsed indication could enhance the differentiation of MC3T3-E1 cells evaluated with pristine PLLA nanofibers. Their outcomes showed that the conductive and biodegradable PLLA/aniline pentamer/gelatin nanofibers had capability of being as bone scaffold materials in tissue engineering in vivo [238]. 

Graphene has obtained exceptional attention in various applications according to its inimitable physical attributes. Lately, several research groups have demonstrated that graphene boosts cellular activities. In 2015, Lyu et al. tested the influence of applying a graphene hydrogel to compel the osteogenic differentiation of hASCs. Compared on arbitrary graphene and carbon fiber films, the hydrogel had enhanced mechanical stability and malleability. Moreover, they discovered that the hydrogel has no cytotoxicity and is biocompatible. One advantage is that film could motive the osteogenic differentiation of hASCs by oneself free of extra chemical signals. Such influences are more powerful for such hydrogel than others; the induction valiancy of the hydrogel is not as high as that of the osteogenic induced medium. The superior osteoinductivity of the hydrogel is nearly linked to its considerable physical attributes that contain special nanoscale structures, surface topology, cell adhesion, surface hydrophilicity, and protein absorption [239]. In 2011, Meng et al. seeded osteoblasts-like Saos-2 cells on an electroactive layer made of PLA and bioactivated PPy using heparin (PPy/HE). The influence of multiple electrical stimulations on osteoblast mineralization was considered at many culture periods using electrical cell culture plates. As confirmed by surface analysis, the electrical stimulation was capable of elevating osteoblast growth and adhesion, causing considerably higher calcium and phosphate concentration in the mineral precipitation of the electrically motivated meshes with similar characteristic features to hydroxyapatite. Electrical stimulation also considerably up ordered the expression of the osteoblasts-specific markers Runt Related Transcription Factor 2 (RRTF-2), Alkaline phosphatase (AP), Bone morphogenetic protein 2 (BMP2), and Osteocalcin. Hence electrical stimulation through a synthetic CPs platform shows a vital factor to enhance bone regeneration [240]. In 2013, Shiyun Meng et al. seeded Saos-2 cells on conductive substrates containing biodegradable PLA and the heparin-PPy to study their response to ES intervened through such scaffolds. Interval and strength of electrical stimulation improved cell proliferation, generating an inimitable electrical intensity and provisional “window” within which osteoblasts proliferation was up-modulated in comparison to the down modulation or ineffectiveness in other electrical stimulation zones. The desirable electrical stimulation intensity around 200 mV/mm was more considered gene activation and protein production of two significant osteoblasts markers described by ECM maturation and mineralization that is AP and osteocalcin [241]. In 2007, Melanie A. Whitehead and Dongmei Fan discussed about of preparation and analysis of an electrically conductive composite material containing PCL, PANi, and silicon. The efficacy of PANi/silicon on calcium phosphate infusion was determined through ex vitro experiment using ES. Formation of calcium phosphate is one conceivable eligible specifications of “intelligent” synthetic scaffolds for orthopedic-pertinent usages. Moreover, electrical consistency evaluations were done in DMEM to assess the constancy of such structures to bias in a reliable electrolyte via a classic cell research. The composites cytocompatibility was measured in vitro via HEK293 cell proliferation, together with more orthopedically pertaining MSCs from mouse stroma. Significantly, these composites show precipitated calcification in simulated body fluid when electrical bias is used catholically to the scaffold. Moreover, these substrates display non-cytotoxicity in the vicinity of fibroblasts during culture period, and annex of stromal cells to the semiconducting scaffold was directly assessed via scanning electron microscopy. Generally, these outcomes propose that such materials are capable to be as a biomaterial [242]. Table 4 abridges the conductive scaffold in bone tissue engineering

### 3.4. Muscular Tissue Engineering

Skeletal muscle tissue includes brindled nanoscale fibrous morphologies convened into fiber fardel which contract upon motivation by an attached nerve [124,252,253,254]. Electrical stimulation has been applied in some clinical trials to considerably help spinal fusion and in the functional recovery and regenerating of muscle in patients who have tolerated denervation. It has been presented that lasting low-frequency electrical stimulation affects myoblast growth and differentiation via duplicating some bioelectric signals [255]. The progression of new tissue engineering experiments for skeletal muscle is serious for the renovation of lost or defective muscle that can happen as a result of traumatic damage or neuromuscular perturbations, such as the Muscular dystrophies [34,252,256]. From a biomimetic outlook, functional engineered skeletal muscle tissues must display indigenous-like structural attributes and, particularly, include compactly packed and uniformly aligned myofibers all over a relevantly large tissue volume [253,254,257]. 

**Figure 10:**
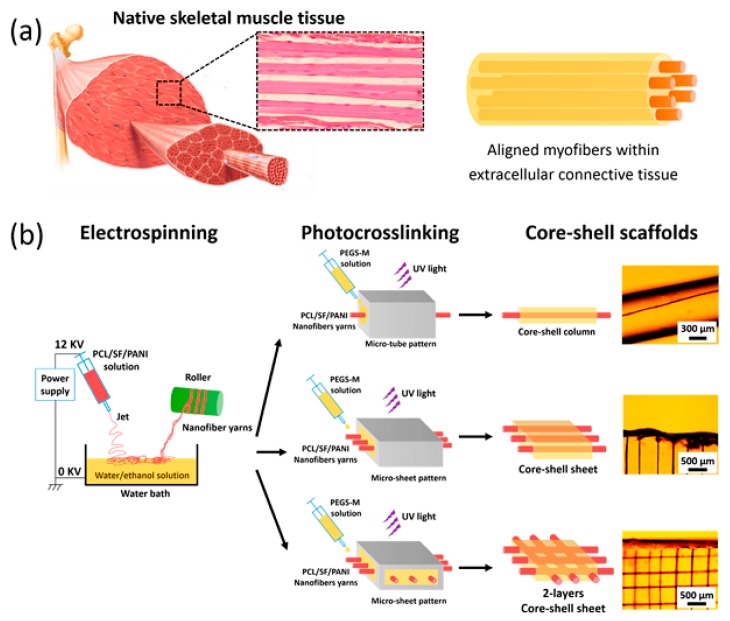
(**a**) Composite similar to the skeletal muscle structure, contain aligned myofibers formed through myoblast fusion together into multinucleated myotubes surrounded within the extracellular connective tissue. (**b**) Scheme of scaffolds fabrication [259], copyright American Chemical Society, 2015.

Another option contains the implantation of prefabricated muscle tissue formed by the in vitro differentiation and puberty of muscle pioneer cells on a matrix or layer. New muscle tissue is expanded in vitro by managing the environmental situations and containing differentiation, with the procedure being seriously related to the material acting as the scaffold for the cells [34,253,256]. Recently, engineering of compact, directed, feigned muscle tissues with a comparatively large area was assayed by consecutively layering collagen matrix and culturing myoblasts in a culture dish [254,257]. One group of materials with potential as a breathtaking candidate for skeletal muscle tissue engineering is CPs. CPs are surmised wonderful as they not only prepare a scaffold for mechanical support, but through their natural conductive attributes can also transfer different stimulation to the cells [34,252,256]. Sung In Jeong and coworkers demonstrated that blending PANi to poly (L-lactide-co-e-caprolactone) scaffold makes ameliorated myoblast cell annex and metabolic activity. The growth of NIH-3T3 fibroblasts is improved under the incitement of several direct current flows between 0-200 mA [75]. In the same study Indong Jun et al. demonstrated that blending of PANi to PLA-PCL scaffold, the number of myocyte cells positive for sarcomeric myosin was 3.6-times higher on the electrically conductive fibers after 4 days of culture. Moreover, the level of myogenin expression deciphered on day 8 of culture on PLA-PCL/PANi-15 (containing 15% PANi) was nearly 1.6-fold greater than the PLA-PCL/PANi-0 fibers. Equivalent outcomes were seen for the expression of other genes containing troponin T (2-fold greater) and the myosin heavy chain gene (3-fold greater) [258]. Wang et al. synthesized the nanofiber yarn/hydrogel core–shell scaffolds for mimicking skeletal muscle (Figure 10). The synthesized scaffold recapitulated the native skeletal muscle tissue which resulted in 3D cellular alignment inducement and elongated myotube formation. The aligned core-shell nanofiber was fabricated by electrospinning based on PCL/PANi/Slik which 3D structure enhanced the nutrient exchange and provided the proper milieu for better alignment and differentiation [259].

A great attention for the engineering of muscle is the capability to restitute tissue in suitable orientation reflecting. Particularly, myotubes should be designed in a linear arrangement to express native muscle structure, which is organized as extremely linear, non-branched bundles in vivo. This organization is in part interposed through the physical and biological attributes of the ECM. The ECM of skeletal muscle includes a nanofibrous network of proteins. Such structure has been repeated ex vivo resulting in the linear orientation of differentiated initial skeletal muscle cells grown on microstructured CP platforms. This efficacy has also been attained at the nanoscale using biodegradable nanofibers, showing that nanoengineered scaffolds prepare the capability to control muscle fiber orientation. The capability to restrain the expansion of myoblasts into orientated myotubes is important for efficient muscle engineering [260]. As an example, Quigley et al. conceived a novel nanostructured conductive scaffold made of aligned MWNT with and without para-toluene sulphonic acid doped PPy. Electrochemical analysis of these substrates demonstrated better electrochemical activity in MWNT after coating. Myoblasts attached, proliferated and differentiated on all collected surfaces sans the use of interpolation molecules. Myotubes grown on nanostructured surfaces showed alignment.

A considerable increment in myotube alignment and length was also discovered on linear functionalized MWNT arrays. The gamut of myotube alignment was discovered to reduce with increasing film thickness. A considerable increment in cell density and myotube formation was distinguished in the electrically motivated group [260]. In two outstanding projects, Ku et al. and Chen et al. divulged that myosin heavy chain expression, multinucleate myotube formation, the emanation of differentiation special genes, the differentiation of myoblasts on PCL/PANi electrospun nanofibers was strongly dependent on both nanofiber alignment and PANi concentration. These outcomes displayed that a composed effect of both guidance cues were more efficacious than a single cue [261,262]. Conductive scaffold which are utilized for muscular tissue engineering is listed in Table 5.

## 4. Conclusions and Future Perspectives

CPs were demonstrated to be able to tune cellular actions via ES such as cell growth and cell migration, leading to a significant interest in CPs and their derivatives for tissue engineering usages. Several research studies are linked to various tissues, which are susceptible to electrical stimulation. This exhibits the significance of CPs in tissue engineering, because the regulation of cellular demeanor is conclusive for the regeneration of blemished tissues. However, there are applied obstacles when the CPs are employed in tissue engineering. The original impediments with the available systems are poor polymer–cell interactions, the absenteeism of cell interaction sites, hydrophobicity, poor solubility and processability, as well as uncontrollable mechanical attributes. Their incapacitation to degrade is one of the greatest constraints for tissue engineering usages. Keeping CPs in vivo for a long time may clasp an inflammatory reaction and the requirement for a second surgical process. The synthesis of materials with both electroactive and degradable attributes is extremely favorable and is still a challenge. There are different fabrication and synthesis paths of biodegradable and electrically CPs using both CPs to form mixes and composites as well as conducting oligomers to form biodegradable and electroactive copolymers [30,265]. 

Provision of clinically appropriate CPs-based tissue scaffolds with biomimetic chemical, mechanical, and topological attributes is the other challenge in this field. These materials can be constructed in a diversity of ways. Biomimetic chemical methods are innovative approaches recently attained that non-covalently combine both high and low molecular weight components/derivatives of the ECM as dopants during electropolymerization reactions. An ECM-mimicking structure can also be exposed to materials by covalently modifying their surfaces with ECM derivatives, generally employing carbodiimide chemistry. Materials composed of CPs only tend to be relevantly inelastic because the polymers have confined conformational freedom in 3D, result in film preparation via electropolymerization rip. This demonstrates a significant problem, as the handling of biomedical products are of key significance to their successful repented from the laboratory to the clinic. Flexible conductive biomaterials can be constructed by interspersing a large-enough quantity of conductive filler within an elastomeric matrix, such as PCL or polyurethane or gathered from multiblock copolymers composed of intermittent blocks of conducting and elastomeric blocks, such as PPy and PCL. The development of tissue engineering scaffolds that mimic wrapped structured natural tissues will be the focus of significant interest in the next few years. Among different strategies, electrospinning is a general method of preparing nanofibrous tissue engineering scaffolds with a tunable degree of fiber alignment nearly similar to the native tissue structure [266].

One of the most challenging issues in tissue engineering is achieving tissue-specific functions. As an example, since hepatocyte cells are anchorage-dependent cells and highly susceptible to the ECM milieu for the keeping of their viability and differentiated functions, liver tissue engineering requires a suitable ECM for hepatocyte cell culture. On the other hand, since initial hepatocytes lose their phenotype quickly after isolation, impounding liver-specific functions has been the main goal of these studies. By implementing innovative physical and chemical strategies, favorable aspects of tissue engineering can be mimic for specific interactions between scaffolds and cell surface receptors. Hence, the design and selection of biomaterials for tissue engineering scaffolds are of great importance. To gain higher levels of tissue-specific function and mechanical consistency, culturing cells have been tested on different conductive biomaterials.

Surface charge and conductivity of biomaterials and different kinds of cells ascertain a communication between the surface voltage, the rest potential, and a control over differentiation and proliferation. It has been determined that the rest potential of cancer and stem cells, which have excellent proliferation rates, are much lower than any kind of adult differentiated cells. After cancer and stem cells, among all the differentiated cells, liver cells are the most depolarized cells within the human body [267]. This phenomenon can be affirmatively influenced in the attendance of conducting biomaterials. In fact, the CPs will conduct the specific charge of the cells that will be resisted in other scaffolds due to resistant materials (e.g., natural polymers) used in the scaffold compositions. The conducted charge can make local electrical fields inside the seeded scaffolds, which will cause regulation of the ion transfer and movement across the membrane and further influence the cellular behaviors, such as cell attachment, cell proliferation, and proteins expression. Some recent studies on hepatocyte cells in tissue engineering scaffolds with different compositions showed that the scaffolds including (PEDOT) could provide the best situation for annex and proliferation of the cells. More particularly, the mixture of hyaluronan, PEDOT, and collagen (I) as dopants in gelatin–chitosan-based scaffolds could introduce the best cell/scaffold actions for regeneration of cells [268,269]. 

The development of innovative biomaterials as structural and bioactive scaffolds is not only essential for tissue engineering but also important for cellular biophysics. Bozhi Tian et al. [270] have recently designed a new type of macroporous nanowire nanoelectronic scaffolds. This class of nanoelectronic scaffolds could mimic the structure of normal tissue scaffolds, organized by self-organization of coplanar reticular networks with built-in strain and by manipulation of 2D mesh matrices. This is one of the important studies showing robust electronic characteristics that have been used lonely or combined with other biomaterials as biocompatible extracellular scaffolds for 3D culture of neurons, CMs and smooth muscle cells [270]. 

The current researches can potentially solve problems associated with the conventional scaffolds that could not electrically probe the physicochemical and biological microenvironments throughout their 3D interior, which can have a noticeable impact in both electronics and biomaterials. Furthermore, the integrated sensory capability of conductive scaffolds by real-time monitoring of the local electrical activity within the constructs could revolutionize the response of neural and cardiac tissue models to drugs, and vital characteristics inside and outside vascular smooth muscle constructs.

## Figures and Tables

**Table 1:** Conductive materials used in tissue engineering.

Conductive Material	Identification Card
**Polyaniline** 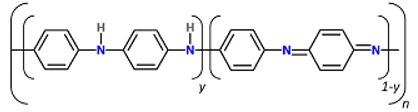	An oxidative polymer with wide ranges of conductivity, cost-effective, easy to synthesis [46]. Max conductivity = 30–200 S/cm
**Polypyrrole** 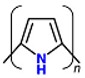	Known for having a wide range of conductivity, insoluble in solvent, and quasi-unidimensional Max conductivity = 40–200 S/cm
**Poly(3,4-ethylenedioxythiophene)** 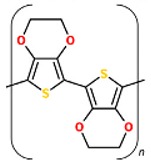	A stable conductive polymer in biological condition with proper biocompatibility [46].
**Polythiophene** 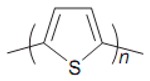	Known for its proper conductivity. Max conductivity = 10–100 S/cm
**Polyacetylene** 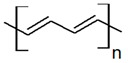	A semi-conducting polymer, which its conductivity can be enhanced using iodine vapor. Polyacetylene was difficult to synthesize and is unstable in air [43].
**Poly (*p*-phenylene)** 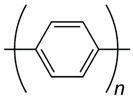	A very high thermally-stable conductive polymer mostly used in rocket nozzles. Its conductivity is 10^2^–10^3^ S/cm.
**Poly(*p*-phenylenevinylene)** 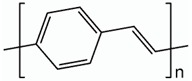	This is the only conducting polymer with highly crystalline thin film formation ability. It can be utilized in photovoltaic devices and light-emitting diodes (LED).
**Poly-*p*-phenylene-sulphide** 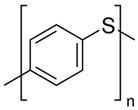	An engineering high-performance thermoplastic, opaque white to light tan in color. It can be molded, extruded, or machined to high tolerances.
**Silicon** 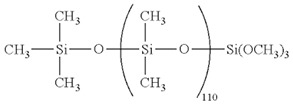	Widely used in chemical and biological sensors and tissue engineering applications. It has controllable electrical conductivity, tunable dimensions, and convenient surface tailorability.
**Melanin** 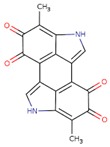	Light-absorbing polymer containing indoles and other intermediate products derived from the oxidation of tyrosine. Melanin is widely found in the animals and plants, and also known as the main pigment in the vertebrates surface structures
**Gold**	Gold has low toxicity, used in drug delivery, imaging and cancer therapy.
**Carbon family**	Carbon allotropes including graphite, diamond and carbon nanotubes show proper mechanical, electrical, thermal, optical, and structural properties [19,47].
**Calcium Titanate**	Calcium titanium oxide is an inorganic compound (CaTiO_3_).

**Table 2:** Conductive scaffolds used in neural tissue engineering.

Conductive Material	Scaffold Type	Scaffold Material	Fabrication Method	Applying Technique	Confirmation Method	Cell Type	Electrical Stimulation/Duration	Major Outcome	Reference
PPy	Film	PPy	Synthesized in two steps by1-(2-cyanoethyl)pyrrole and NHS-1-ethyl-3-(3-dimethylaminopropyl)	Electrochemically polymerized	Enzyme-linked immunosorbent assay	PC12	No	External electrical potential to NGF-immobilized PPy films did not cause a significant release of NGF nor reduce their neurotrophic activity	[157]
		PPy	Ring-opening polymerization	Electrochemically polymerized	Cyclic Voltammetry	Rat C6 cells	Yes/square wave, frequency of 1 Hz, 5% duty cycle, and electrical potential of 0.1 V	Differentiation of rat neuronal pheochromocytoma PC-12 cells	[158]
		PPy/chitosan	Micro emulsion polymerization	Blending	-	Schwann cells	Yes/a lateral constant potential gradient (100 mV/mm, 4 h)	Composite dramatically enhanced the expression and secretion of NGF and BDNF	[142]
		PPy/poly(3,4-ethylenedioxythiophene)	Electrochemical polymerization	Coating	Cyclic Voltammetry (CV)	Neural cells	No	A RC parallel circuit must be added to the model for PEDOT + live neuron and neuron-templated PEDOT coatings.	[159]
		PPy	Polymerized galvanostatically	Polymerized galvanostatically	-	hNSCs	8 h per 24 h period for 3 days	Use of electrical stimulation to promote neuronal induction and the biocompatibility of PPy(DBS) with hNSCs	[160]
		PPy/poly(ε-caprolactone)-poly(lactic acid) (PLA)	Polymerization of pyrrole on electrospun PCL or PLA	Coating	-	Dorsal root ganglia	No	The results suggest the potential use of the conductive coresheath nanofibers as scaffolds in applications such as neural tissue engineering	[161]
		PPy/PCL Fumarate	Pre-formed PCLF scaffolds	polymerizing pyrrole in pre-formed PCLF scaffolds	4-point probe method	PC12-dorsal root ganglia	Yes/DC current source	PCLF-PPy materials synthesized with NSA or DBSA support cell attachment, proliferation, neurite extension	[50]
		PPy/(PCLF)	All chemicals were purchased from Fisher or Aldrich and used as is unless noted otherwise.	blending	-	PC12-	Yes/1 h/day of 10 μA 20 Hz	significant increases in percentage of neurite bearing cells, number of neurites per cell	[45]
		PPy/chitosan	in-situ polymerization	in-situ preparation	Electromyography	neonatal rat cardiomyocytes	No	PPy/chitosan synchronized the contraction of physically-isolated cardiomyocytes clusters without external electrical stimulation.	[162]
		PPy/chitosan	Freeze–drying method.	Mixing	Masson Trichrome Staining	Left sciatic nerve	Yes/intermittent ES (3 V, 20 Hz)	Establishing an electrical environment with electrical stimulation localized at the conductive scaffold is capable of accelerating nerve regeneration and promoting functional recovery in nerve defect in rats	[163]
		PPy/poly (d,l-lactide) (PDLLA)	PDLLA was purchased from Boehringer Ingelheim (Germany)	Mixing	Cyclic Voltammetry	PC12 cells	Yes/100 mV for 2 h	When the PPY/PDLLA nerve conduit was used to repair a rat sciatic nerve defect it performed similarly to the gold standard autologous graft.	[164]
		PPy/silk fibroin solution-PDMS	soft lithography technique	Coating	Cyclic Voltammetry	NGCs	No	Dorsal root ganglions adhere to the films and the grooves in the surface of the films instruct the aligned growth of processes extending from the Dorsal root ganglions	[165]
	Hydrogel	PPy/PHEMA–MMA	-	Blending	EDL muscle mass and EDL maximal specific muscle force	Peroneal nerve gaps	Yes/current density of 0.5 mA cm for 30 min	The PEDOT lining may be used to facilitate future studies using electrical stimulation and/or controlled release of neurotrophins. In addition to promoting axonal growth, the conductive lining may be used as an effective interface between the electronic circuitry of neural prosthetic devices and the peripheral nervous system	[166]
	Nanofibers	PPy/PLLA	Electrospinning	Coating	-	Pc12	Yes/100 mV/cm voltages through the composite fibers.	PPy-PLLA fibers could support PC12 neurite outgrowth and extension	[167]
		PPy/PLGA	Electrospinning	Coating	-	PC12	No	Stimulation of the cells on aligned PPy-PLGA fibers resulted in longer neurites and more neurite-bearing cells than stimulation on random PPy-PLGA fibers, suggesting a combined effect of electrical stimulation and topographical guidance	[143]
		PPy/Gelatin- poly(*ε*-caprolactone)	Acidic condition selectrospining	Mixing	-	C17.2-neural cells	No	The use of electrically CP very attractive for the construction of scaffolds for nerve tissue Engineering.	[168]
		PPy/poly(3-hydroxybutyrate-co-3-hydroxyvalerate)/799 po ly(e-caprolactone) (PHBHV/PCL	Electrospinning	Mixing	-	NGCs	No	The current trend of peripheral nerve tissue engineering is the design of advanced nerve guidance channels acting as physical guidance for regeneration of nerves across lesions. Nerve guidance channels should present multifunctional properties aiming to direct the sprouting of axons from the proximal nerve end, to concentrate growth factors secreted by the injured nerve ends, and to reduce the ingrowth of scar tissue into the injury site.	[169]
		PPy/poly(lactic acid)	Electrospinning	Coating	Two-Point Probe	Neural progenitor cells	Yes/steady potential of 100 mV for 2 h continuing with biphasic 100 mV for 4 h per day, for three days	PPy-coated electrospun PLA fibers had a good biocompatibility with neural progenitor Cells	[170]
Carbon	Film	Carbon nanotube (CNTs)	-	-	Cyclic Voltammetry	Neural cells- glial cells- Schwann cells	No	Accumulating data support the use of CNTs as a biocompatible and permissive substrate/scaffold for neural cells and such application holds great potential in biomedicine	[171]
		Graphene/Polyelectrolyte	Layer-by-layer (LbL) deposition	-	Probe analysis	Primary cortical neurons (PCNs)	No	Electro active scaffold modification may therefore assist in neuronal regeneration, for creating Functional and biocompatible polymer scaffolds for electrical entrainment or bio sensing applications.	[172]
		Graphene	Pre-fabricated	-	Cyclic voltammetry	NSCs	Monophasic cathodic pulses with stimulation threshold was 20–30 μA	3D-GFs can enhance the NSC differentiation towards astrocytes and especially neurons	[30]
		CNT/PLDLA	Chemically tethered onto the surface	Mixing	Cyclic Voltammetry	Rat sciatic nerve	Yes/5 mA stimulus intensity, 1 Hz frequency, 1 ms duration	In vivo effect of using a CNT-interfaced scaffold in the regeneration process of a transected rat sciatic nerve strongly supports the potential use of CNT-interfaced PGFs at the Interface between the nerve conduit and peripheral neural tissues.	[140]
		Graphene/PCL	Conventional electrospinning process	Mixing	-	PC L 12- mMSCs	No	Hybridization of GO nanosheets and PCL polymer dramatically enhanced the differentiation of the mMSCs and PC12-L cells into osteo- and neuro-like cells www.win	[173]
		carbon nanotube (CNT)/polyvinyl alcohol (PVA) -(PVA-polypyrrole)	Casting technique to a silicone mould	Coating	-	Mesenchymal stem cellS (MSCS)	No	Results revealed that treatment with MSCs and PVA-CNTs tube-guides induced better Nerve fiber regeneration.	[174]
	Nanofibers	carbon nanofiber (CNF)/PLLA	Electrospinning	Blending	-	mESC	Yes/frequency range of 1–106 Hz.	Conductive scaffold could be a useful tool for the generation of neural Tissue mimics in vitro and potentially as a scaffold for the repair of neural defects in vivo.	[155]
		CNF	-	-	-	Human epidermal keratinocytes (HaCaT)	No	Possibility of utilizing carbon nanostructures to repair a long gap in nerve	[175]
		CNT	-	-	Using a Multiclamp 700B amplifier	Neonatal rat spinal cord	Electrophysiological recordings were acquired using a Multiclamp 700B amplifier (Molecular Devices), sampled at 10 kHz and digitized by a Digidata 1440A analog-to-digital converter.	CNT-incorporated/supported platforms trigger reparative activities involving microglia, in the absence of reactive gliosis	[176]
		CNT/PLLA	Electrospinning	Mixing	Cyclic Voltammetry	OEC	No	SWNT/PLLA nanofibrous scaffolds and OEC could promote axonal outgrowth and peripheral nerve regeneration	[177]
		CNT/poly(3,4-ethylenedioxythiophene)-	Electrospinning	Coating	Cyclic Voltammetry	Motor neurons	Yes/100 mV pulse electrical signal	GO sheets could be fabricated into 3D fine size nanofibers scaffold by the controlled assembly of GO sheets. The obtained G-NFs exhibited recoverable electrical conductivity, soft Acceptable physical characteristic and excellent biocompatibility and physicochemical stability. An unprecedented accelerated growth and development of the primary motor neurons was achieved by using the G-NFs for cellular electrical stimulation in a long-term culture period.	[178]
	Hydrogel	Carbon nanotube	High-pressure carbon monoxide conversion synthesis	High-pressure carbon monoxide conversion synthesis	-	Schwann cell (SC)-glial	No	An electrically-conductive SWNT collagen biomaterial may be suitable for neural tissue engineering and is able to sustain populations of SC.	[179]
		CNT/collagen	Mixing	Coating	Four-point probe	Pc12	No	Enhanced electrical activity and have shown positive in vitro biocompatibility results offering further evidence that SWNT-based materials have an important role in promoting neuronal regeneration.	[180]
		CNT/PEDOT	-	Coating	-	-	No	Organic conductors provide safe electrical stimulation of tissue while avoiding undesirable chemical reactions and cell damage.	[181]
PANi	Nanofibers	PANi/poly (ɛ-caprolactone/gelatin)	Electrospinning	Coating	-	Nerve stem cells	1.5 V for 15, 30, and 60 min	Conductive nanofibrous scaffolds are suitable substrates for the attachment and proliferation of nerve stem cells.	[182]
		PANi/PLLA	Electrospinning	Blending	-	Nerve stem cells	Steady potential of 1.5 V for a period of 60 min	implication of electrical stimulation of nerve stem cells on conducting polymeric scaffolds towards neurite elongation that could be effective for nerve tissue regeneration	[21]
		PANi/PLCL	Electro spinning	Blending	4-point probe	Pc12	No	Electro active fibers may hold Promise as a guidance scaffold for neuronal tissue engineering.	[183]
		PANi/PCL	Ring-opening polymerization	Blending	4-probe technique	HaCaT cell line	No	These scaffolds are non-cytotoxic. These degradable electroactive tubular scaffolds are good candidates for neural tissue engineering application	[184]
		PANi	Oxidation of aqueous solutions of aniline by ammonium peroxydisulfate (APS) at 30 °C	Oxidation	-	Human mesenchymal stem cells	Yes/electric field (DC)was applied for 10 min, and the same sequence was repeated again over an interval of 24 h	The present work establishes the key role of intermittent and systematic delivery of electric stimuli as guidance cues in promoting neural-like differentiation of hMSCs, when grown on electroconductive Substrates.	[144]
	Hydrogel	PANi/PEGDA	UV photopolymerization	Blending	2-probes	PC12-hMSC	Yes/AC current amplitude of 0.01 mA over a frequency range from 1 to 20 × 10^3^ Hz	PANi also improve the biological response of PC12	[146]
		PANi/Collagen	Chemical synthesis	Coating	4-point probes	PC12	No	PANI and PEDOT nanofibres were found to be cytocompatible with both cell types and the best results were obtained with a low Concentration (0.5 wt%) of PANI.	[185]
		PANi/Chitosan/Gelatin	Stirring	Mixing	4-point probe	Schwann cells from infant human sciatic nerves	No	Porous conductive chitosan/gelatin/PAG scaffold having proper conductivity	[186]
PEDOT	-	Decellular nerve scaffolds	Electrochemical method	Coating	Cyclic voltammetry	-	No	Low concentrations of PEDOT on Decellular nerve scaffolds provided significant increases in electro active properties	[187]

“-”: N/A.

**Table 3:** Conductive scaffolds used in cardiovascular tissue engineering.

Conductive Material	Scaffold Type	Scaffold Material	Fabrication Method	Applying Technique	Confirmation Method	Cell Type	Electrical Stimulation/Preferences	Major Outcome	Reference
PPy	Film	PPy	electrochemically polymerized	Electrochemically polymerized	4-probe technique	HUVEC	No	This bioactive conductive platform provides a functional surface capable of tethering biomolecules that direct cell behavior without the drawback of reduced conductivity.	[217]
		PPy	Electrochemical synthesis of PPy film then doped with HA and coated with collagen IV followed by Matrigel	Electrochemical	Cyclic voltammetry	Vascular smooth muscle cell	Yes/50 mA sinusoidal, 0.05, 5 and 500 Hz for 24 and 96 h	Vascular smooth muscle cells (VSMCs) cultured on a CP substrate and subject to electrical stimulation not only exhibit enhanced proliferation but can be simultaneously encouraged to increase contractile protein expression.	[215]
		PCL/PPy	Heat-pressed	Chemical polymerization	4-probe technique	Artrial myocyte	No	When CMs were cultured on the electrically-conductive PPy-PCL, more cells were observed to have peripheral localization of the gap junction protein connexin-43 (Cx43). Importantly, the velocity of calcium wave propagation was faster, and calcium transient duration was shorter for CM monolayers on PPy-PCL relative to cells on PCL.	[210]
	Nanofibers	PCL/gelatin/PPy	Elecrospinning	Co-elecrospinning	Surface resistivity by standard four-probe method	Rabbit Cardiomyocyte	No	By increasing the concentration of PPy in the composite, the average fiber diameters reduced, and the tensile modulus increased. In addition, this scaffold promote cell attachment, proliferation, interaction, and expression of cardiac-specific proteins.	[192]
	Porous scaffold	PPy	Electropolymerization deposition within a sacrificial agarose gel template	Electropolymerization	4-probe technique	HUVEC	No	The porous-structured PPy increased the viability of HUVECs. The higher viability of HUVECs on the porous-structured PPy can be attributed to the increased surface area and three-dimensionally hollow micro-/nanostructures, which can facilitate cell distribution and adhesion.	[218]
	Hydrogel	Alginate/PPy	Bivalent crosslinking	Blending	4-probe technique	HUVEC	No	Addition of PPy mediated cell attachment and proliferation. local injection of polymer blend in to the infarct zone yielded significantly higher levels of arteriogenesis at 5 weeks post-treatment. Also, this scaffold significantly enhanced infiltration of myofibroblasts into the infarct area.	[216]
PANi	Film	PANi	Heating and vacuum drying	Heating and vacuum drying	Surface resistivity by standard four-probe method	H9c2	No	Both conductive and non-conductive form of PANi improve cell attachment and proliferation in comparison with Polystyrene plate.	[219]
		PCL	Solvent casting	Blending and nanoneedles	picometer/voltage source meter	hMSC and cardiomyocyte-like cell	No	PANi short fibers provide a more efficient transfer of electric signal. this electrically conductive environment are able to stimulate the cell differentiation to cardiomyocites, for a successful use in the myocardium muscle regeneration.	[203]
		PU containing aniline pentamer/PCL	Casting	PU containing aniline pentamer	4-probe technique	HUVEC and L929	No	Bioelectroactive polyurethane is a platform substrate to study the effect of electrical signals on cell activities and to direct desirable cell function for tissue engineering applications.	[71]
		poly(glycerol-sebacate)/PANi	Solvent casting	Blending	4-probe technique	C2C12	No	The novel PANi–PGS composites, being able to maintain their electrical conductivity, not only have the potential to modulate cellular function but, when fabricated in 3-D porous scaffolds, also hold promise to serve as carrier and delivery vehicles of functional cells to the myocardial infarct.	[220]
	Nanofibers	Gelatin/PANi	Elecrospinning	Co- elecrospinning	Potentiostat/Galvanostat	H9c2	No	By increasing the amount of PANi the average fiber size reduced and the tensile modulus increased. This scaffold supported cell attachment and proliferation.	[38]
		Nanofibers contained PANi nanotubes modified by polyglycerol dendrimers	Electrospinning	Electrospinning	-	Cardiac myocytes and CHO cell	No	The PGLD-coated PANINTs showed noncytotoxic effects to Chinese hamster ovary cells. It was observed that the application of microcurrent stimulates the differentiation of cardiac cells cultured on these scaffolds.	[194]
		Composite nanofibers of PANi and PLGA	Electrospinning	Co-elecrospinning	4-probe technique	Neonatal cardiomyocyte	No	During incubation, the adhered CMs became associated with each other and formed isolated cell clusters; the cells within each cluster elongated and aligned their morphology along the major axis of the fibrous mesh.	[209]
	Porous scaffold	PU containing aniline pentamer/PCL	Compression molding	PU containing aniline pentamer	4-probe technique	Neonatal cardiomyocyte	No	Scaffolds supported neonatal CMs adhesion and growth, showing more extensive effect on the expression of the cardiac genes involved in muscle contraction and relaxation and cytoskeleton alignment.	[221]
Carbon	Film	PLGA-CNF composite	Heating and vacuum drying	Blending	Multimeter	Cardiomyocyte and neuron	No	PLGA:CNF materials are conductive, and that the conductivity is related to the amounts of CNF. Also, CM and neuron density increases with greater amounts of CNF in PLGA.	[190]
		PLGA-CNF composite	Heating and vacuum drying	Blending	Multimeter	Human cardiomyocyte	No	Results showed that PLGA:CNF materials were conductive. Furthermore, results indicated that CM density increased with greater amounts of CNFs of 200nm in diameter in PLGA.	[222]
		PLGA-CNF composite	Heating and vacuum drying	Blending	-	Aortic endothelial, 3T3 and cardiomyocyte	Yes/rectangular, 2 nm, 5 V/cm, 1 Hz for 24, 72 and 120 h	This composite can stimulate CM growth and activity while hindering fibroblast and endothelial cell growth. During continuous electrical stimulation, CM cell density increased in comparison to its static counterparts after 24, 72 and 120 h. A minor rise in Troponin I excretion in electrical stimulation compared to static conditions indicated nominal CM cell function during cell experiments.	[208]
		MWNT	Deposition/drying to have a thin film	Deposition/drying	-	Neonatal rat ventricular myocyte	No	The proliferative capacity of CMs on carbon nanotubes was significantly higher. Cells grown on CNT substrates displayed values of resting potential more negative than those of gelatin cells.	[205]
		MWNT	Deposition/drying to have a thin film	Deposition/drying	AFM with a Veeco Nano Scope V in tapping mode	Neonatal rat ventricular myocyte	No	CNT scaffolds promote CM growth and maturation by altering the gene expression program, implementing the cell electrophysiological properties and improving networking and maturation of functional syncytia.	[206]
		Collagen/SWNT	Deposition/Vacuum drying	Dispersion	Multimeter	Neonatal cardiomyocyte	No	The addition of CNTs remarkably increased intercalated disc related protein expression and enhanced ID assembly and functionality. Notably, CNTs remarkably accelerated gap junction format ion via activation of the b1-integrin-mediated FAK/ERK/GATA 4 pathway.	[210]
	Nanofibers	PU/MWNT	Electrospinning	Co-elecrospinning	-	HUVEC	No	Experimental results indicated that the nanofibrous scaffold of MWNT/PU exhibited promotional influence on the cell proliferation. It was also observed that the scaffold possessed an advantage of supporting Endothelial Cells migrating and aggregating along the axis of the aligned nanofibers. It was also demonstrated that the endothelial cells growing on the scaffold expressed non-thrombogenic phenotype with low tissue factor released.	[223]
		PLA/SWNT	Electrospinning	Co-elecrospinning	Electrical resistance measure	hMSC	Yes/0.15 V/cm for 2 ms duration at a frequency of 1 Hz	After electrical stimulation the cells reoriented perpendicular to the direction of the current and adopted an elongated morphology. An upregulation in a range of cardiac markers was detected.	[224]
		poly(e-caprolactone)/MWNT	Electrospinning	Co-elecrospinning	Electrochemical impedance spectroscopy	hMSC	Yes/500 v/m and 5 ms pulse width at 1 Hz, for 10 min per day for 4 days	hMSC differentiation can be enhanced by either culturing in electrically conductive, carbon nanotube-containing composite scaffolds without electrical stimulation in the presence of 5-azacytidine, or extrinsic electrical stimulation in nonconductive poly(e-caprolactone) scaffolds without CNT and azacytidine.	[204]
		poly(glycerolsebacate)/gelatin/CNT	Electrospinning	Co-elecrospinning	Electrochemical Impedance spectrometry	Cardiomyocyte	No	Aligned CNT-PG scaffold exhibited superior mechanical properties with enhanced CM beating properties.	[196]
	Porous scaffold	Chitosan/CNF	Precipitation	Dispersion	Picoammeter -Voltage source	Neonatal rat cardiomyocytes	No	Incorporation of CNFs into porous chitosan scaffolds improved the properties of cardiac tissue constructs, presumably through enhanced transmission of electrical signals between the cells.	[225]
		Collagen/SWNT	Gelation at 37 °C	Dispersion	-	Rat aortic smooth muscle cells (RASMC)	No	Cell viability in all constructs was consistently above 85% at both Day 3 and Day 7.	[226]
	Hydrogel	pHEMA/rosette nanotubes/CNF	Radical polymerization	Dispersion	Multimeter	Cardiomyocyte	No	CM density increased after 4 h, 1 day, and 3 days with greater amounts of CNFs and greater amounts of RNTs in pHEMA. In fact, wettability, conductivity, and surface nanoroughness become greater with greater amounts of CNFs and RNTs.	[188]
		Gelatin/SWNT	Glutaraldehyde crosslinking	Dispersion	Multimeter	Neonatal rat cardiomyocytes	No	SWCNTs could provide cellular microenvironment in vitro favorable for cardiac contraction and the expression of electrochemical associated proteins. Upon implantation into the infarct hearts in rats, this scaffold structurally integrated with the host myocardium.	[212]
		Gelatin methacrylate/CNT	Photopolymerization under UV light	Dispersion	Electrochemical Impedance spectrometry	Neonatal rat cardiomyocytes	No	myocardial tissues cultured on 50 µm thick CNTGelMA showed 3 times higher spontaneous synchronous beating rates and 85% lower excitation threshold, compared to those cultured on pristine GelMA hydrogels.	[199]
		Poly (N-isopropylacrylamide) modified with SWNT	In situ forming (gelled above 32 °C)	Dispersion	Electrochemistry workstation	Brown adipose-derived stem cell	No	In vitro study showed that the PNIPAAm/SWNTs hydrogel demonstrated significantly higher bioactivities to encapsulated brown adipose -derived stem cells compared with onefold PNIPAAm hydrogel, including promoting cell adhesion and proliferation. When used as carrier for intramyocardial delivery of BASCs after MI, the PNIPAAm/SWNTs hydrogel significantly enhanced the engraftment of seeding cells in infarct myocardium and augmented their therapeutic efficacies in MI.	[207]
		gelatin/chitosan/SWNT	Lyophilization	Dispersion	Voltage sensitive dye and an ionoptix system	Ventricular myocyte	No	These engineered tissues achieve excitation conduction velocities similar to native myocardial tissue and could function as a full-thickness patch for several cardiovascular defect repair procedures.	[227]
Au	Nanofibers	Polyvinyl alcohol/Bovine serum albumin/Au nanoparticles	Electrospinning	Dispersion	-	hMSC	No	AuNPs loaded nanofibrous scaffolds facilitates the functional differentiation of MSCs.	[123]
	Hydrogel	HEMA/Au nanoparticles	Photopolymerization under UV light	Dispersion	Electrometer	Neonatal rat cardiomyocyte	No	Neonatal rat CMs exhibited increased expression of connexin 43 on hybrid scaffolds relative to HEMA with or without electrical stimulation.	[36]
		Alginate/Au nanowire	Bivalent crosslinking	Dispersion	Electrochemical Impedance spectrometry	Neonatal rat cardiomyocyes and fibroblasts	No	Incorporating gold nanowires within alginate scaffolds can bridge the electrically resistant pore walls of alginate and improve electrical communication between adjacent cardiac cells. So thicker and better aligned tissue grown on this scaffold and cells in these tissues contracted synchronously.	[211]
melanin	Nanofibers	Poly(L-lactide-co-3-caprolactoe)/gelatin/melanin	Electrospinning	Co-elecrospinning	4-probe technique	Human cardiac myocyte	Yes/rectangular, 150 ms, 1 V/cm, 1 Hz for 4 and 8 days	Conductive nanofibers containing 10% melanin promote cell interaction with expression of cardiac-specific proteins compared to other scaffolds. Electrical stimulation through the scaffolds showed enhanced cell proliferation and the expression of connexin-43.	[132]
silicon	Hydrogel	Agarose/silicon nanowire	Casting	Dispersion	-	Rat neonatal cardiac cells and human induced pluripotent stem cell	No	Incorporation of a trace amount of electrically conductive silicon nanowires (e-SiNWs) in otherwise scaffold-free cardiac spheroids can form an electrically conductive network, leading to synchronized and significantly enhanced contraction, resulting in significantly more advanced cellular structural and contractile maturation.	[119]
**PEDOT**	Porous scaffold	poly(styrenesulfonate)/PEDOT	Ice-templating	Physical crosslinking	Electrochemical transistor	3T3 cell	No	The scaffolds support the growth of mouse fibroblasts for 7 days, and are able to electrically control cell adhesion and pro-angiogenic capability. These 3D matrix-mimicking platforms offer precise control of protein conformation and major cell functions, over large volumes and long cell culture times.	[228]

“-”: N/A.

**Table 4:** Conductive scaffolds used in bone tissue engineering.

Conductive Material	Type	Composition	Fabrication Method	Applying Technique	Confirmation Method	Cell Type	Electrical Stimulation/Duration	Major Out Come	Reference
PPy	Film	PPy/Polypropylene fumarate	Rapid prototyping technique	Coating	-	Osteoblasts	No	Osteoblasts maintained their phenotype on PPF scaffolds with and without coatings. Thus, these Scaffolds could be appropriate candidates for our future in vivo studies	[243]
		PPy/PLLA/Heparin	Water-in-oil emulsion	Blending	-	Osteoblasts-like Saos-2 cells	Yes/200 mV/mm 6h per day	The electrical stimulation was able to promote osteoblast adhesion and growth, resulting in significantly higher calcium and phosphate content in the mineral deposition of the electrically stimulated membranes.	[240]
		PPy/PLA	Extruding	Coating	-	Human adipose stem cells	Yes/DC voltage repeated at a frequency of 1 or 100 Hz, ES for 4 h/day.	The alkaline phosphatase (ALP) activity of the hASCs was generally higher in PLA-PPy seeded scaffolds	[244]
		PPy/poly(L-lactide)	Chemical synthesis	Blending	-	Saos-2 cells	Yes/Four potential intensitieswere applied to the conductive membranes, that is 100, 200, 300, and 400 mV/mm. The cells were stimulated for 2, 4, 6 and 8 h at each ES intensity	This work demonstrated that important osteoblast markers can be modulated with specific electrical stimulation parameters mediated through CPs substrates, providing a unique strategy for bone tissue engineering.	[241]
		PPy/Hyaluronic acid or chondroitin sulfate	Chemical synthesis	Coating	-	Human adipose stem cells	Yes/Samples were stimulated for 4 h a day for 14 days with a biphasic electric current (BEC) of ± 0.2 V amplitude, 2.5 ms pulse width and 100 Hz pulse repetitionFrequency.	PPy–chondroitin sulfatein particular is a potential osteogenic scaffold Coating for bone tissue engineering.	[230]
Carbon	Film	CNT/PCL	Salt-leaching technique	Coating	-	Human kidney fibroblasts cells	Yes/Varying voltages (0.5 and 0.7 V) were applied in a cathodic direction to the scaffold in medium	Materials of this type of composition have potential merit as a biomaterial	[242]
		CNT/poly(ester amide)s	Chemical synthesis	Mixing	Cyclic Voltammetry	Preosteoblastic MC3T3-E1	Yes/The square wave, frequency of 50 Hz, 50% duty cycle, And electrical potential of 0.2 V was adopted in the experiment.	The PEA-g-TA copolymers stimulated by pulsed electrical signal could serve to promote the differentiation of MC3T3-E1 cells	[245]
		Carbon nanotube	-	-	-	Osteoblasts, chondrocytes smooth muscle cells and fibroblasts	No	Studies are evaluated with an emphasis on understanding the mechanisms through which 17 carbon nanotubes interact with biological systems.	[246]
		Graphene/collagen	Chemical synthesis	Coating	-	MC3T3-E1	No	Scaffolds modified with a suitable concentration of GO are useful as a bioactive material for tissue engineering	[247]
		Graphene/citrate-stabilized HA hydrocolloids	Self-assembly	mixing	Cyclic Voltammetry	Mouse multipotent mesenchymal stromal cells	No	The resulting graphene-HA gels are highly porous, strong, electrically conductive and Biocompatible, making them promising scaffolds for bone tissue engineering. This method can be applied to produce a variety of free-standing 3D graphene-based nanocomposites with Unprecedented homogeneity.	[248]
		Graphene/TCP	Oxidation	Coating	-	BMMSCs	No	Combination of graphene and goat mesenchymal stemcells provides a promising construct for bone tissue engineering.	[249]
	Hydrogel	Graphene/Chitosan	Chemical synthesis	-	Cyclic Voltammetry	L-929	No	These chitosan-graphene composites show great promise for use as conducting substrates for the growth of electro-responsive cells in tissue engineering.	[250]
PANi		PANi/PGA-g-TA/PLL-g-TA	Ring-opening polymerization	Coating	-	Preosteoblastic MC3T3-E1	Yes/The square wave, frequency of 100 Hz, 50% duty cycle, and electrical potential of 0.5 V were adopted	The comprehensive effects through coupling electroactive scaffolds with electrical stimulus are better to develop bioelectric strategies to control cell functions for bone regeneration	[236]
		PANi/PLLA	Electrospinning	Coating	Cyclic Voltammetry	Preosteoblastic MC3T3-E1	Yes/The square wave with frequency of 50 Hz, electrical potential of 0.2 Vand 50% duty cycle was adopted. The samples were stimulated for 2 h every day, respectively.	Biodegradable and electroactive AP-g-GA/PLLA nanofibers had potential application in vivo as bone repair scaffold.	[238]
		PANi/PCL/gelatin composite	Electrospining	Mixing	Cyclic Voltammetry	-	No	By incorporation of conductive PANi and bioactive particles and drugs (osteogenon, calcium phosphate nanoparticles) within an electrospun PCL/gelatin composite scaffold, we have obtained a biocompatible, bioactive, hybrid scaffold system, which provides an electrically conductive environment.	[251]
PEDOT	Film	PEDOT/poly(3,4-ethylenedioxythiophene) poly(4-styrene sulfonate)	Chemical synthesis	Blending	-	Human mesenchymal stem cells	No	Conductive scaffolds are not only structurally more favorable for bone tissue engineering, but also can be a step forward in combining the tissue engineering techniques with the method of enhancing the bone healing by electrical stimuli.	[237]

“-”: N/A.

**Table 5:** Conductive scaffolds used in muscular tissue engineering.

Conductive Material	Scaffold Type	Scaffold Material	Fabrication Method	Applying Technique	Confirmation Method	Cell Type	Electrical Stimulation/Preferences	Major Outcome	Reference
PANi	Nanofibrous	Poly(L-lactide-co-e-caprolactone)/PANi	Electrospinning	Mixing	4-probe technique	Human dermal fibroblasts, NIH-3T3 and C2C12 myoblasts	Yes/DC 0–200 mA	Addition of PANi to scaffold causes improved cell attachment. In addition, the growth of NIH-3T3 fibroblasts is enhanced under the stimulation of various direct current flows. The incorporation of PANi improved the metabolic activity of all cell types treated in a concentration-dependent manner.	[75]
		Poly(L-lactide-co-3-caprolactone)/PANi	Electrospinning	Mixing	4-probe technique	C2C12 myoblasts	No	The prepared PLC L/PANi fibers showed no significant difference in fiber diameter or contact angle, regardless of the incorporation of PANi.after 4 days of culture, the number of cells positive for sarcomeric myosin was 3.6-times greater on the electrically conductive fibers.	[258]
		Polycap rolactone/PANi	Electrospinning	Mixing	Potentiostat/galvanostat	C2C12 myoblasts	No	Myosin heavy chain expression, multinucleate myotube formation, and the expression of differentiation specific genes, the differentiation of myoblasts on PCL/PANi nanofibers was strongly dependent on both nanofiber alignment and PANi concentration.	[262]
		polycap rolactone/PANi	Electrospinning	Mixing	4-probe technique	C2C12 myoblasts	No	The aligned nanofibers could guide myoblast orientation and promote myotube formation. In addition, electrically conductive nanofibers further enhanced myotube maturation compared with non-conductive scaffolds.	[261]
		Chitosan grafted aniline tetramer	Electrospinning	Mixing	Cyclic voltammetry	C2C12 myoblasts	No	The chitosan grafted aniline tetramer substrates and their degradation products are not cytotoxic and could improve the cell adhesion and proliferation of C2C12 myoblasts compared to chitosan.	[82]
		Tetraaniline-polylactide	Thermally induced phase separation	Blending	Cyclic voltammetry	C2C12 myoblasts	No	These electroactive degradable materials are nontoxic and enhance the adhesion and proliferation of the C2C12 myoblast cells compared to polylactide, probably because of the more proteins adsorbed on the electroactive materials than that of polylactide. The electroactive materials significantly improved the cell proliferation of C2C12 myoblasts under ES.	[263]
	Hydrogel	Gelatin–graft–polyaniline	Crosslinking by genipin	Grafting	Cyclic voltammetry	C2C12 myoblasts and MSC	No	The conductivity of this insitu forming degradable hydrogel in the swollen is proportional to PANi content in the materials. The non-cytotoxicity of the hydrogels was confirmed via cell adhesion and proliferation.	[64]
		Chitosan-graft-PANi/Oxidized Dextran	In situ forming	Grafting	Cyclic voltammetry	MSC and C2C12 myoblasts	No	This anitibacterial hydrogel had a fast mass loss in the first 5.5 weeks with a linear degradation trend. After that time, the degradation speed of the hydrogels went slowly and still showed linear degradation kinetics. in addition, with the increase of PANi content, the dead cell were less and they exhibited a higher cell proliferation. Furthermore, this hydrogel could form in short time by injection in vivo.	[67]
PPy	Film	PPy doped with ECM components	Galvanostatically deposition	Galvanostatically deposition	Gamray Impedance system	Skeletal muscle myoblasts	No	Polymer films including PPy/HA and PPy/para-toluene sulphonic acid showed good support for myoblast proliferation but were poor in terms of adhesion and differentiation. In contrast, PPy/poly (2-methoxy-5 aniline sulphonic acid) supported a lower degree of proliferation, but good cell adhesion and differentiation.	[256]
PPy/MWNT	Aerogel sheet	MWNT	Deposition	Deposition	-	Murine primary muscle cell	Yes/0.125 mA/cm^2^ biopolar square wave, 10 Hz, 8 h per day for 3 days	Application of electrical stimulation to myoblasts on nanostructured MWNT/para-toluene sulphonic acid doped PPy platforms led to significant enhancements in total myo-nuclear density and myoblast differentiation.	[260]
carbon	Nanofibrous	Polyurethane/CNT	Electrospinning	Mixing	4-probe technique	C2C12 myoblasts	Yes/22 V/cm, 20 Hz, biphasic, twice a day on the last 2 days of culture	After electrical stimulation, the number of multinucleated myotubes on the electrospun polyurethane CNT scaffolds was significantly larger than that on nonconductive electrospun polyurethane scaffolds. In the absence of electrical stimulation, myoblasts also differentiated on the electrospun polyurethane CNT scaffolds, as evidenced by expression of Myf-5 and myosin heavy chains.	[252]
		Styrene/butadiene/styrene/CNT	Electrospinning	Mixing	slope of I–V curves measured with an automated Keithley 487 picoammeter/voltage source	C2C12 myoblasts	No	With the introduction of CNT in the polymers, the Styrene/butadiene/styrene samples proved to be cytotoxic contrarily to styrene–ethylene/butylene–styrene samples. The styrene–ethylene/butylene–styrene composites are thus a suitable candidate for biomedical applications, including the development of scaffold membranes for tissue engineering applications.	[264]
PTh	Film	Poly(octanoic acid 2-thiophen-3-yl-ethyl ester)	Spin coating	Spin coating	Cyclic voltammetry	Primary skeletal muscle myoblasts and C2C12 myoblasts	No	The polymer films supported the proliferation and differentiation of both primary and transformed skeletal muscle myoblasts. In addition, aligned electrospun fibers formed from the polymers provided scaffolds for the guided differentiation of linearly aligned primary myotubes, suggesting their suitability as three-dimensional substrates for the in vitro engineering of skeletal muscle tissue.	[34]
Au	Nanofibrous	Poly(L-lactic acid)/Au nanoparticles	Electrospinning	Mixing	Measuring electrical resistance	Rat primary muscle cell	No	The first cell study showed low cell proliferation on the Au–PLLA scaffolds; however, the second cell study showed that this was not due to Au Nps toxicity. Instead, low cell proliferation may be a marker for myotube differentiation and fusion. By electrospinning higher amounts of Au Nps with PLLA, a conductive, biocompatible and biodegradable scaffold can be manufactured for skeletal muscle tissue engineering that could possibly use lower voltages to increase myotube formation.	[124]

“-”: N/A.

## Abbreviations

ECM	extracellular matrix
SCI	spinal cord injuries
NDD	neurodegenerative diseases
CM	cardiomyocyte
MI	Myocardial infarction
hASCs	human adipose stem cells
MSC	mesenchymal stem cell
NGF	Nerve growth factor
BDNF	Brain-derived neurotrophic factor
CP	conductive polymer
PPy	polypyrrole
PANi	polyaniline
XCA	xanthan hydrogels
PLA	poly(lactic acid)
PEDOT	poly (3, 4-ethylenedioxythiophene)
PLLA	poly(L-lactic acid)
PDLLA	poly (d,l-lactide)
CNT	carbon nanotube
SWNT	single wall carbon nanotube
MWNT	multiple wall carbon nanotube
CNFs	carbon nanofibers
3D-GFs	three-dimensional graphene foams
PCL	polycaprolactone
AuNPs	Gold nanoparticles
SiNWs	Silicon nanowires
HA	hyaluronic acid
PNIPAAm	poly (N-isopropylacrylamide)
SGH	self-supporting graphene hydrogel
PABS	poly (aminobenzene sulfonic acid)
